# ‘Ribozoomin’ – Translation Initiation from the Perspective of the Ribosome-bound Eukaryotic Initiation Factors (eIFs)

**DOI:** 10.2174/138920312801619385

**Published:** 2012-06

**Authors:** Leoš Shivaya Valášek

**Affiliations:** Laboratory of Eukaryotic Gene Regulation, Institute of Microbiology AS CR, Videnska 1083, Prague, 142 20, the Czech Republic

**Keywords:** Translation initiation, ribosome, eIF, mRNA, AUG, translational control, reinitiation, GCN4.

## Abstract

Protein synthesis is a fundamental biological mechanism bringing the DNA-encoded genetic information into life by its translation into molecular effectors - proteins. The initiation phase of translation is one of the key points of gene regulation in eukaryotes, playing a role in processes from neuronal function to development. Indeed, the importance of the study of protein synthesis is increasing with the growing list of genetic diseases caused by mutations that affect mRNA translation. To grasp how this regulation is achieved or altered in the latter case, we must first understand the molecular details of all underlying processes of the translational cycle with the main focus put on its initiation. In this review I discuss recent advances in our comprehension of the molecular basis of particular initiation reactions set into the context of how and where individual eIFs bind to the small ribosomal subunit in the pre-initiation complex. I also summarize our current knowledge on how eukaryotic initiation factor eIF3 controls gene expression in the gene-specific manner via reinitiation.

## INTRODUCTION

Translation is the final step of the Central Dogma in Molecular Biology capturing the flow of genetic information in the cell. Translational control critically contributes to the overall regulation of gene expression, development, stress responses, memory formation and aging. Compared to transcriptional regulation, translational control of existing mRNAs allows for more rapid changes in cellular concentrations of the encoded proteins and, thus, can be used for maintaining homeostasis, in addition to modulating more permanent changes in cell physiology or fate. Indeed, there are numerous examples demonstrating that deregulation of translational control either directly causes various diseases or significantly contributes to their rapid development (for example neurodegenerative conditions, diabetes, cancer, etc.). Translation can be divided into initiation, elongation, termination, and ribosome recycling, with the initiation phase serving as the primary target of the most regulatory pathways (reviewed in [1]). Hence it is not surprising that the recent decade or two have experienced a robust wave of studies exploring the molecular basis of every initiation substep that can be regarded as a potential point of control. 

## TRANSLATION INITIATION IN EUKARYOTES

**Fig. (1):**
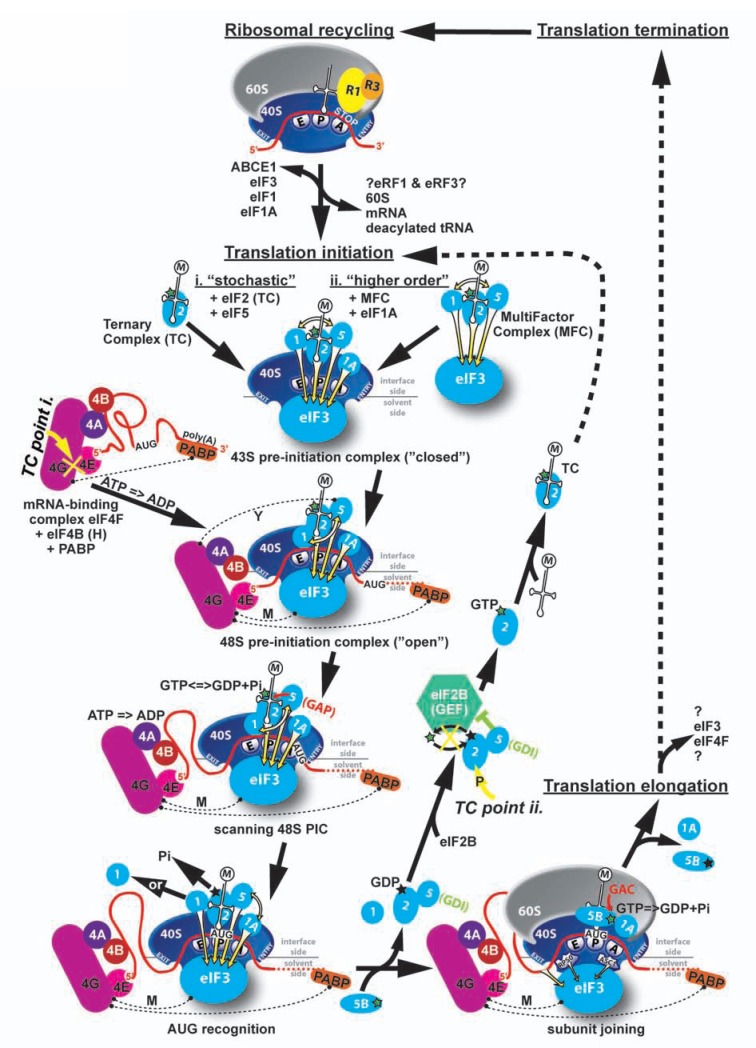
Schematic of the canonical translation pathway in eukaryotes with the ribosomal recycling and initiation phases shown in detail. This figure combines findings from both yeast and mammals and indicates potential differences. The terminating 80S ribosome is split into individual subunits with help of ABCE1/RLI1 and eIFs 1, 1A and 3. How eRFs 1 and 3 are recycled is not properly understood. The former eIFs either remain bound to the 40S subunit or dissociate prior to the initiation phase. In the former case, the Met-tRNAi ^Met^•eIF2•GTP ternary complex (TC) and eIF5 join the existing 40S-eIF1-eIF1A-eIF3 post-recycling complex in a “stochastic” way (i) to form the 43S pre-initiation complex (PIC). In the latter case, the 43S PIC is formed in the “higher order” manner via simultaneous binding of all components of the Multifactor complex (eIFs 1, 3, 5 and the TC) and eIF1A. Upon binding, eIFs1 and 1A induce conformational change that opens the mRNA binding channel of the 40S ribosome for mRNA loading. As a part of this major rearrangement eIF1, if delivered to the ribosome in the MFC, must translocate from eIF3 to the P-site. mRNA is delivered to the 43S PIC in a complex with eIF4F (composed of eIF4A, E and G), eIF4B (and/or eIF4H in mammals) and PABP in an ATP-dependent reaction creating a “landing pad” close to the mRNA’s cap structure that is bound by eIF4E (the interaction between eIF4G and PABP is shown as a dotted line for simplicity). As a result, the 48S PIC is formed and scanning for AUG commences. The actual attachment of mRNA to the ribosome is believed to be mediated via the eIF4G – eIF3 interaction in mammals (dotted line “M”) that seems to be bridged via eIF5 in yeast (dotted line “Y”; this line is not shown in all cartoons for simplicity). During scanning, all secondary structures that could impede the movement of the PIC in the 5' to 3' direction are melted with help of helicase eIF4A and its co-factors eIF4B or eIF4H at the expense of ATP. Also, eIF5 stimulates GTP hydrolysis on eIF2 (GAP activity), however, the resulting Pi is not released until the AUG is located. Upon AUG recognition, eIF1 as a gatekeeper is either ejected from the ribosome or could move back to eIF3 to allow Pi release triggering reciprocal conformational switch to the closed form of the PIC that arrests scanning. eIF5B then promotes subunit joining that kicks out all interface-side-bound eIFs with the exception of eIF1A, and the solvent-side-bound eIF3 and eIF4F (interactions between eIF3 and two “solvent-side” ribosomal proteins RPS0 and RACK1/ASC1, based on [41,59,117], are indicated). GTP hydrolysis on eIF5B stimulated by the GTPase activating center (GAC) of the large subunit triggers coupled release of eIF5B and eIF1A rendering the resulting 80 initiation complex ready to elongate. It is believed that eIF3 and eIF4F can stay 80S-bound for at least a few elongation cycles thanks to their location on the back of the 40S subunit. eIF2•GDP is released in a binary complex with eIF5 that competes with and thus partially inhibits the action of the GEF eIF2B to exchange GDP for GTP on eIF2 (GDI activity). Upon this exchange, eIF2•GTP is ready to form a new TC that can enter the entire cycle all over again. See text for more details. Two “Translational control (TC) points” briefly mentioned in the main text are indicated by yellow arrows and the mechanism of their action by yellow cross lines; the first targets the eIF4E–eIF4G interaction and the other the GTP/GDP exchange on eIF2 by phosphorylating its α subunit.

Canonical eukaryotic translation initiation ensures timely and spatially coordinated formation of the trimeric complex between the 40S small ribosomal subunit (40S), initiator Met-tRNA_i_^Met^ and an mRNA at its extreme 5’ end, and concludes with the assembly of an elongation-competent 80S ribosome at the authentic AUG start codon (summarized in Fig. **1**). The entire process is orchestrated by individual proteins and three protein complexes commonly called eukaryotic initiation factors (eIFs). To begin a new translational cycle, first a pool of separated ribosomal subunits has to be generated from those that have just finished (terminated) translation of a given mRNA in the „previous“ cycle. The ultimate product of translation termination that has to be recycled into individual components is called a post-termination ribosomal complex (post-TC). It consists of an 80S couple still bound to mRNA, P-site deacylated tRNA and eukaryotic release factors (eRFs) 1 and 3 (or at least eRF1). Based on experiments carried out with purified mammalian factors in *in vitro *reconstituted systems it was originally proposed that, at low Mg^2+^ concentration (Mg^2+^ ions promote ribosomal subunit association), recycling can be mediated solely by eIFs 1, 1A and 3 [2]. In detail, eIF3, together with its loosely associated eIF3j subunit, eIF1 and eIF1A, first dissociates 60S subunits from the post-TCs. Subsequently, eIF1 promotes release of the tRNA from the P-site of the liberated 40S subunits. Finally, eIF3j significantly enhances eIF3’s mRNA dissociation activity to complete the recycling reaction. However, later on it was reported that with elevated Mg^2+^ concentrations, recycling strictly depends on ABCE1 (ATP-binding cassette subfamily E member 1), which ensures splitting of post-TCs into free 60S subunits and tRNA•mRNA•40S complexes in the first recycling reaction [3] (Fig. **1**). Consistently, RLI1, a yeast homologue of ABCE1 was also shown to be involved in termination *per se *in *S. cerevisiae*, however, its precise role remains to be elucidated [4]. Preliminary results likewise indicate that yeast eIF3 directly participates in translation termination and/or recycling (L. Cuchalová, P. Beznosková, T. von der Haar, and L.S.V., unpublished observations). Hence, it appears that the role of ABCE1/RLI1 and several canonical initiation factors as “terminators” of the translational cycle is conserved among eukaryotes. It should be noted that the way how eRFs 1 and 3 are ejected from post-TCs is still unclear. Finally, eIF6, a protein associated with the 60S subunit, is believed to prevent ribosomal subunit re-association [5-7]. 

In the first step of a new translational cycle, Met-tRNA_i_^Met^ is bound by the trimeric eIF2 complex in its GTP form to produce the Met-tRNA_i_^Met^•eIF2•GTP ternary complex (TC). Subsequently, the multiprotein eIF3 complex, together with eIFs 1, 1A and 5, promotes recruitment of the TC to the small ribosomal subunit (40S), producing the 43S pre-initiation complex (PIC) (reviewed in [8-10]). In fact, numerous studies carried out over the last two decades suggest that there are two major ways of how eIFs associate with the ribosome to form the 43S PIC: i) the “stochastic – prokaryotic-like” pathway with eIFs binding to the small subunit on individual basis; and ii) the “higher order – eukaryotic” pathway, where eIFs 1, 3, 5 and the TC assemble into a large multifactor complex (MFC) and approach the 40S ribosome as a pre-organized unit (for example [11-20]) (Fig. **1**). Importantly, recent data from plant [21] and human [22] cells provide evidence that these two pathways are evolutionary conserved among all eukaryotes. By definition, the MFC-driven pathway is generally considered to ensure the efficiency of the whole initiation process especially under conditions permissive for growth. In any case, upon initial binding of the aforementioned factors, eIFs 1 and 1A serve to stabilize a specific conformation of the 40S head relative to its body that opens the mRNA binding channel for mRNA loading. That requires dissolving the latch formed by helices 18 (h18) and 34 (h34) of 18S rRNA and establishing a new interaction between RPS3 and h16 [23].

In the next step, in the current text book view, eIF3 and the eIF4F complex promote recruitment of mRNA to thus “activated” 43S PIC with help of the poly(A)-binding protein (PABP) forming the 48S PIC. eIF4F comprises the cap-binding protein eIF4E, the DEAD-box RNA helicase eIF4A and eIF4G, which functions as a “scaffold“. It binds eIF4E, eIF4A, PABP and in mammals also eIF3, through which the connection between the eIF4F•mRNA and the 43S PIC could be bridged (Fig. **1** – “M” dashed line). In budding yeast, direct eIF3-eIF4G interaction has not been detected, and the eIF3-binding domain is not evident in yeast eIF4G [24]. Instead it was proposed that eIF5 might bridge the contact between eIF4G and eIF3 in the 48S PIC, as it was shown to be capable of simultaneous binding to both factors *in vitro* [25] (Fig. **1** – “Y” dashed line). Taking into account that yeast eIF3 is also considered to be more critical factor for mRNA recruitment than eIF4G [15,20], it could be that the molecular mechanism of this particular initiation step differs in certain aspects between lower and higher eukaryotes. Alternatively, in the light of the recent *in vivo *studies carried out in yeast and mammalian cells, it seems also plausible that the mRNA recruitment step is, in general, less dependent on the direct eIF4G–eIF3 contact than it has been believed so far (see below) [26-29]. Importantly, stable binding of the 43S PIC near the 7-methylguanosine cap of natural mRNAs requires melting the secondary structures that often occur in their 5’ untranslated region (UTR) and the eIF4A helicase, as part of the eIF4F complex, is the prime candidate for this role. It should also be mentioned that formation of an interaction between the cap-binding protein eIF4E and eIF4G has been shown to serve as one of the two major targets for the general translational control, especially in mammalian cells (Fig. **1**) (reviewed in [1]). In yeast the global controls that feed off this regulatory step have not been clearly identified as yet indicating that they might not be so robust. 

In contrast to prokaryotic cells, the mRNAs of which posses a Shine-Dalgarno sequence ensuring a direct placement of the start codon into the ribosomal P-site, eukaryotic ribosomes have to search the 5' UTR of an mRNA for usually the first AUG codon by a successive movement called scanning [30]. This is accompanied by unwinding secondary structures in an ATP-dependent reaction stimulated by helicases eIF4A (with its co-activators eIF4B or eIF4H), DHX29 and DED1 (reviewed in [10]) (Fig. **1**). The mechanism of scanning *per se* is still largely unexplored. Besides the requirement for helicases, it was shown that in the absence of secondary structures, the presence of the TC and eIFs 1, 1A, and 3 in 48S PICs suffices for locating the AUG start in the mammalian reconstituted systems [31]. 

Most importantly, during scanning ribosomes have to read, integrate and respond to a variety of poorly understood signals that orchestrate the AUG recognition process (reviewed in [32]). These signals originate from mutual molecular and functional interactions between mRNA and the 40S ribosome with a number of initiation factors such as eIF1, eIF1A, eIF2 (TC), and eIF5. In the open conformation of the 40S ribosome that is induced by eIFs 1 and 1A, as mentioned above, and that is conducive for scanning, the anticodon of Met-tRNA_i_^Met^ is not fully engaged in the ribosomal P-site in order to prevent premature engagement with putative start codons. eIF2 partially hydrolyzes its GTP with the help of the GTPase accelerating factor (GAP) eIF5; however, prior to start codon recognition, the “gate-keeping” function of eIF1 prevents the release of the resultant phosphate ion, producing GTP- and GDP•Pi-bound two states of the factor, possibly in equilibrium [33]. Encounter of the AUG start codon induces a reciprocal conformational switch of the 48S PIC to the closed/scanning arrested form, stabilized by a functional interaction between eIF1A and eIF5 [34], with the initiator Met-tRNA_i_^Met^ fully accommodated in the P-site [35]. This irreversible reaction serves as the decisive step stalling the entire machinery at the AUG start codon and is triggered by dissociation or displacement of eIF1 [36], possibly promoted by eIF1A and eIF5, and subsequent release of free Pi (Fig. **1**). In short, eIF1 and eIF1A (via its C-terminal tail) antagonize the codon-anticodon interactions in the P-site by blocking the full accommodation of initiator tRNA in the P-site in a manner that is overcome efficiently by the action of the N-terminal tail of eIF1A and eIF5 upon establishment of a perfect AUG-anticodon duplex in an optimal Kozak AUG context [37]. As will be discussed later, besides the aforementioned factors, there is an increasing number of reports suggesting that also the multifunctional eIF3 complex significantly contributes to the regulation of AUG recognition [11,14,17,19,38].

The scanning-arrested 48S PIC can now join the large ribosomal subunit with the help of GTP-bound eIF5B [39,40], upon which most of the interface-side-based eIFs are ejected with the exception of eIF1A, and most likely also eIF3 and eIF4F [41-43]. Finally, GTP-hydrolysis on eIF5B stimulated by the GTP-ase activating center (GAC) of the 60S subunit triggers the release of eIF1A and eIF5B producing an active 80S ribosome poised for elongation (Fig. **1**).

To enter a new initiation cycle, “discharged” eIF2•GDP must interact with the pentameric eIF2B, which acts as the GTP/GDP exchange factor (GEF) for eIF2 and exchanges its GDP for a GTP nucleotide [44,45]. Only this „charged“ form of eIF2 can stably bind Met-tRNA_i_^Met ^to form a new ternary complex. According to the recent reports, eIF2•GDP leaves the PICs in the binary complex with eIF5 that antagonizes eIF2B-promoted guanine nucleotide exchange (see below) [46,47] (Fig. **1**). It is important to note that the step of the ternary complex formation is the other of the two major targets of the general translational control (Fig. 1) (reviewed in [1]). Several kinases phosphorylate the α-subunit of eIF2 upon various cellular stress conditions turning it form a substrate to an inhibitor of an exchange reaction, which leads to a global translational shut down (reviewed in [48]).

## SMALL RIBOSOMAL SUBUNIT (40S) – the CENTRAL HUB

**Fig. (2):**
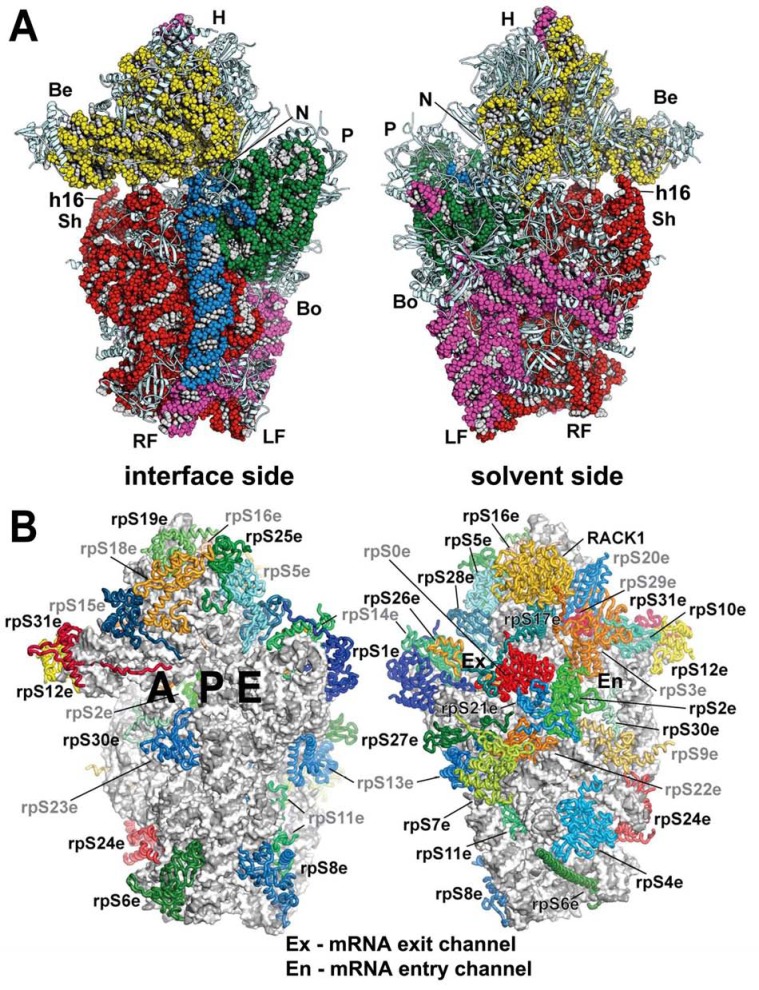
Architecture of the crystal structure of the 40S subunit (adapted from [52]). (**A**) Interface and solvent-exposed views of the tertiary structure of the 40S showing the 18S rRNA as spheres and colored according to each domain (5' domain, red; central domain, green; 3' major domain, yellow; 3' minor domain, blue; ESs, magenta), and the proteins as gray cartoons (abbreviations: H, head; Be, beak; N, neck; P, platform; Sh, shoulder; Bo, body; RF, right foot; LF, left foot). (**B**) Ribosomal proteins of the 40S are shown as cartoons in individual colors; rRNA is shown as gray surface. The 40S is shown in the same orientation as in (**A**).

Whereas peptide bond synthesis occurs at the large ribosomal subunit, the small ribosomal subunit is responsible for decoding the information encoded in mRNA (reviewed in [49]). Relative to the bacterial small ribosomal subunit (30S), the eukaryotic small ribosomal subunit (40S) is larger by almost 500 kD. It consists of 33 proteins, 18 of which do not have homologs in bacteria, and an 18S rRNA (versus the bacterial 16S rRNA) [50,51]. Many of the additional functions of eukaryotic ribosomes involve the small ribosomal subunit because of its critical role in translation initiation. One of the novel aspects is that it has to accommodate far more initiation factors, and possibly also various regulatory molecules, than its bacterial counterpart. Recently, three crystal structures of the eukaryotic ribosomes were reported as major advances in the field [52-54], one of which featured specifically the small ribosomal subunit of *T. thermophila* in complex with eIF1 (Fig. **2**). This structure defined the locations and the folds of all 33 eukaryotic ribosomal proteins as well as all eukaryotic expansion segments (ESs) of 18S rRNA, and the details of their mutual interactions [52]. For the purpose of this review I will highlight only a few features of the 40S subunit that will become relevant later on.

- The beak of the eukaryotic ribosome has transformed from an all-rRNA structure to a protein protuberance in eukaryotes: Eukaryotic ribosomal proteins RPS10, RPS12, and RPS31 are bound to a reduced h33 of the 18S rRNA, giving the eukaryotic protein beak essentially the same conserved shape as observed for the bacterial 30S subunit [52].
- Helix h16, which is situated directly below the beak (Fig. **2A**), is together with RPS3 involved in forming a connection between the head and the body of the 40S subunit upon binding of initiation factors eIF1 and eIF1A that opens up the mRNA binding channel for mRNA recruitment [23].
- The mRNA binding channel in eukaryotic 40S bears similar characteristics to that of 30S; however, individual features have been extensively remodeled. Several basic residues of eukaryotic RPS3 (homolog to bacterial rpS3p) as well as of an extension of the eukaryotic-specific RPS30 are oriented toward the mRNA channel and could thus interact with the phosphate backbone of the mRNA secondary structure, which is unfolded by movement of the head relative to the body [55].
- The yeast ribosomal protein ASC1 and its mammalian ortholog RACK1 are both members of the WD40 (Trp-Asp) repeat scaffold protein family that adopt a seven-bladed β-propeller structure. RACK1/ASC1 (designated as ASC1 henceforth) is located on the head of the 40S ribosomal subunit near the mRNA exit tunnel and makes extensive contacts with helices h39 and h40 of 18S rRNA and ribosomal proteins RPS16, 17, and 3 [52,56]. ASC1 was also shown to interact with a number of signaling molecules on and off the ribosome and thus it was proposed to play an important role in a multitude of biological processes and to serve as a regulatory link between signaling and translation (reviewed in [57]). For example, mammalian RACK1 recruits activated protein kinase C to the ribosome, which leads to the stimulation of translation through the phosphorylation of eIF6 [6]. In a ribosome-free form, RACK1 associates with membrane-bound receptors [58], which could be instrumental for docking the ribosomes to sites where local translation is required, such as focal adhesions. However, direct experimental evidence for the latter is missing. Its contribution to the initiation process *per se *has been unclear until very recently, when it was demonstrated that it promotes assembly of the 43S pre-initiation complexes by making a direct contact with eIF3 [59] (see below). It should also be stressed here that although there are numerous reports ascribing various phenotypes to the null allele of non-essential yeast *ASC1*, many of these reports worked not only with the deletion of the *ASC1* coding sequence *per se*, but also with the deletion of the *ASC1 *intron carrying *SNR24* encoding the C/D box U24 snoRNA [60]. We and others recently showed that deletion of *SNR24 *affects cellular growth on its own as it impairs 60S biogenesis and produces halfmers [59,61]. (Halfmers are formed by mRNAs containing elongating 80S ribosomes and the 48S PICs stuck in the mRNA leader). Under these circumstances it seems difficult to predict what of the reported phenotypes were directly associated with the deletion of *ASC1 *only.

### eIF1 – the GATEKEEPER

**Fig. (3):**
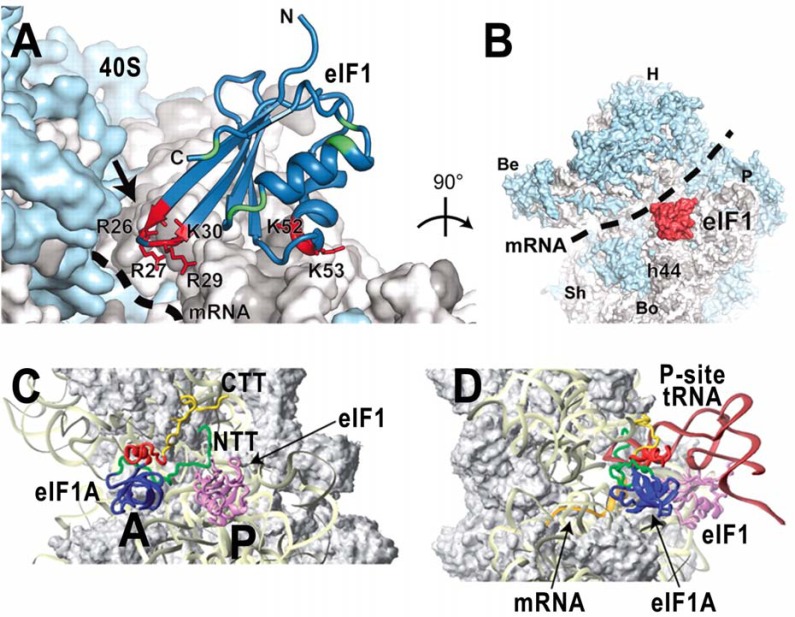
(**A - B**) Crystal structure of eIF1 in complex with the 40S subunit (adapted from [52]). (**A**) Eukaryotic initiation factor eIF1 binds to the 18S rRNA phosphate backbone (shown as gray surface) with basic residues (shown in red). The basic loop of eIF1 found in close proximity to the mRNA channel is indicated with an arrow. Genetic experiments implicate residues behind the basic loop, at the end of one of the helices and the penultimate residue in cognate codon recognition (green). (**B**) T. thermophila eIF1 (red) is located at the top of h44 below the platform. Dashed lines indicate the mRNA path on the 40S subunit. (**C - D**) Mutual orientations of eIF1A, eIF1, mRNA and ^Met^-tRNAi Met on the 40S subunit (adapted from [75]). Comparison of the positions of eIF1A (shown in blue, red, yellow and green) and eIF1 (violet ribbon) on the 40S subunit. Note that although the modeled position of eIF1A-NTT appears in proximity to eIF1 in this view, the two do not contact each other. (**D**) View of the mutual orientation of eIF1A, eIF1, mRNA, and ^Met^-tRNAi Met on the 40S subunit, rotated 90° clockwise, compared to panel C. Ribosomal protein S13/RPS18 in the head is not shown as it blocks the view of a portion of eIF1A-CTT.

eIF1 is a 12 kDa protein that consists of a single α/β domain and an unstructured N-terminal region [62]. It is functionally analogous to the C-terminal domain of bacterial IF3 [63] and, accordingly, it has been shown, using site-directed hydroxyl radical probing, to bind in a similar position close to the P-site of the 40S subunit [64]. The recent crystal structure of the *Tetrahymena *40S–eIF1 complex [52] confirmed in great detail that basic residues in helix α1 and the β1-β2 loop of eIF1 interact with residues in helices 44 and 24 of 18S rRNA in the platform region of the 40S subunit (Fig. **3A - B**). The unstructured N-terminal tail (NTT) of eIF1 mediates its binding to the N-terminal domain (NTD) of eIF2β and the C-terminal domain (CTD) of eIF5 *in vitro *and reduces the rate of TC recruitment, yet it is dispensable for yeast viability [36]. eIF1 is critically involved in the scanning and AUG recognition processes. Upon binding to the 40S, it is, together with eIF1A, thought to evoke a conformational switch from the closed/scanning arrested state to the open state of the “latch” of the mRNA entry pore, in which the positions of mRNA and loosely bound initiator tRNA on the ribosome are conducive to scanning but incompatible with start codon selection [23,31]. eIF1 was also shown to antagonize the Met-tRNA_i_^Met^ anticodon interactions with near-cognate triplets or with AUG in the suboptimal initiation context during scanning in a manner that is overcome efficiently only with a perfect AUG-anticodon duplex in an optimal AUG context and with the 5‘ UTR long enough to interact extensively with the 40S mRNA binding channel [31,65]. Rabl *et al.* suggested that actually the basic residues of eIF1 in the loop between β1 and β2 might be responsible for monitoring the quality of the codon-anticodon duplex as they appear close enough to the mRNA-binding channel [52] (Fig. **3A - B**). 

Furthermore, eIF1 is believed to serve as a gatekeeper as it prevents the release of Pi from eIF2-GDP•Pi prior to the AUG start codon recognition [66]. A biochemical analysis of eIF1 in the mammalian reconstituted system by Pestova and colleagues provided evidence that eIF1 additionally restrains the GAP function of eIF5 at non-AUGs, an activity that would reduce the formation of eIF2-GDP•Pi in addition to blocking Pi release in the scanning complex [67]. Based on biophysical studies conducted with the yeast *in vitro *reconstituted system using eIFs 1, 1A, 5 and TC but not eIF3, eIF1 was proposed to be ejected from PICs upon AUG recognition [66]. However, an alternative, purely hypothetical option is that upon AUG recognition, eIF1 triggers a reciprocal conformational rearrangement from the open to closed/scanning arrested state and drifts back to the c/NIP1-NTD in the A-site as an integral part of this conformation change (see also below). This eIF1 translocation or its ejection, initiated potentially by its clash with the acceptor and D stems of initiator tRNA dwelling on AUG [52] and powered most probably also by eIF5 [68,69], is thought to “open the gate” for the subsequent irreversible Pi release resulting in the scanning arrest. In any case, the release of Pi upon AUG recognition evokes rapid and highly stable Met-tRNA_i_^Met^ binding in the P-site [70] fixing the AUG-anticodon interaction, which disables further inspection of successive triplets entering the P site during scanning. More stable Met-tRNA_i_^Met^ binding in the PICs with eIF1 either ejected or just away from the P-site area may sound contradictory to the long known fact that both eIF1 and eIF1A collaborate to increase the rate of TC recruitment (see for example [71]). However, it was shown by the Lorsch lab that the affinity of the TC for the PIC is actually lower in the presence of eIF1, owing to eIF1’s greater stimulation of the TC dissociation rate than the association rate [36,68]. 

Interestingly, Ivanov *et al.* [72] recently noticed that all genes encoding eIF1 in eukaryotes contain an AUG in a poor Kozak consensus context [37]. Experimental analysis of this finding revealed that mammalian eIF1 autoregulates its own translation, and regulates translation in general, by discriminating against poor AUG context *in vivo*, as suggested from the aforementioned *in vitro *experiments. The authors showed that overexpression of eIF1 in mammalian cells had a negative effect on eIF1 expression with AUG in the poor (genuine) but not in the optimal Kozak context (see also below).

### eIF1A – the AUG TAILor

eIF1A is a 17 kDa protein that possesses a β-barrel OB-fold domain, a short additional helix together with long extended N-terminal and C-terminal tails (NTT and CTT) [73]. It is a sequential and structural homolog of bacterial IF1 and, accordingly, its globular OB domain binds to the A-site in reconstituted mammalian 43S complexes as IF1 does to the A-site of the 30S subunit [74,75] (Fig. **3C - D**). Whereas the OB domain most probably anchors eIF1A on the ribosome, both of its unstructured tails are critically required for multiple functions that eIF1A ensures such as stimulation of the PIC assembly, scanning, and AUG recognition [31,76-79]. The CTT contains two 10 amino acid direct repeats dubbed scanning enhancer 1 (SE1) and SE2 [35] that were shown to stabilize an open conformation of the 40S subunit and thus to promote scanning and a mode of TC binding that blocks the full accommodation of initiator Met-tRNA_i_^Met^ in the P site (so called P-out state). Consistently, in the docking model, the eIF1A-CTT was predicted to extend out into the P-site, threading under the Met-tRNA_i_^Met^ in a manner that could obstruct the tight binding of the initiator tRNA anticodon stem loop in the canonical location in bacterial 70S elongation complexes [75] (Fig. **3C - D**). In contrast to the CTT, the eIF1A-NTT – predicted to contact the anticodon loop of initiator Met-tRNA_i_^Met^ directly [75] – contains the scanning inhibitor (SI) element that is together with the helical domain thought to promote eIF1 dissociation or displacement when AUG enters the P-site, and to stimulate ejection of the SEs from the P-site to permit the full accommodation of initiator Met-tRNA_i_^Met^ (so called P-in state) [35]. Furthermore, it was proposed that thus ejected eIF1A-CTT functional interacts with eIF5 in a way that reduces its flexibility and strengthens eIF1A binding to the PIC [34]. In other words, the action of the CTT-based SEs during scanning is, upon AUG-anticodon base-pairing, antagonized by the NTT-based SIs to arrest scanning by switching to and stabilization of a closed conformation of the 40S subunit. Accordingly, the 40S-eIF1A cryo-EM complex, which may mimic this situation shortly after the Pi release when the latch firmly clamps down on the mRNA, displays a more closed conformation of the latch than that which occurs for the apo-40S [23].

### eIF2 – the MET-tRNA_i_^Met^ DELIVERYMAN

eIF2 is a heterotrimer, comprised of α, β, and γ subunits, which together form a ~120 kDa complex. Its primary functions are selection and recruitment of Met-tRNA_i_^Met^ to the 40S ribosomal subunit in form of the eIF2•GTP•Met-tRNA_i_^Met^ ternary complex and control of the start site recognition. eIF2 might, for example, contribute to the different modes of Met-tRNA_i_^Met^ binding to the P site that are thought to characterize the open and closed conformations of the PIC, as mentioned above. eIF2 interacts with Met-tRNA_i_^Met^ in its eIF2•GTP state, which shows roughly 10-fold higher affinity for Met-tRNA_i_^Met^ than the eIF2•GDP state [80,81]. GTP hydrolysis or loss of the methionine moiety thus weakens the interaction of eIF2 with the initiator Met-tRNA_i_^Met^, which is the critical step of the AUG selection process. Since the release of GDP from the γ subunit is very slow (~0.2 min^-1^), the resulting eIF2•GDP needs to be recycled to eIF2•GTP by the GEF eIF2B in order to be able to form a new ternary complex (Fig. **1**) [44,45,82]. This requirement provides critical means for one of the two general mechanisms of translational control, as suggested above. Interestingly, the basal GTPase activity of eIF2 is very low and is significantly increased only when the TC becomes a part of the PIC [33]. This indicates that it likely depends on some structural features in eIF2 that couple its GTPase activity to interactions with the 40S subunit components. Also, in eukaryotes, but not in archaea, GTP hydrolysis requires the action of its GAP factor in eIF5 (see below). Remarkably, the first nucleotide base-pair of Met-tRNA_i_^Met^ (A1:U72) serves as the main determining factor for its specific and stable interaction with eIF2•GTP [83,84] and is essential to orient the charged methionine into its binding pocket on the γ subunit [81]. Recent work using *S. cerevisiae* has identified a specific nucleotide of the 18S rRNA (A928), located within the P-site, that is required for loading and affinity of the ternary complex on the 40S subunit perhaps by making a direct contact with the initiator Met-tRNA_i_^Met^ [85]. Even though the eIF2•GTP•Met-tRNA_i_^Met^ ternary complex can bind stably to the 40S ribosomal subunit on its own, this interaction is greatly stabilized by other factors such as eIFs 1, 1A and eIF3 (reviewed in [8]).

Whereas practically no complex structures have been obtained for any of the eukaryotic eIF2 subunits, X-ray crystal structures were solved for the archaeal aIF2γ protein, both free and in complex with full-length or truncated versions of aIF2α and aIF2β, but always in the absence of Met-tRNA_i_^Met^ [86-95]. The full complex structure revealed highly flexible α and β subunits, which are expected to be stabilized upon binding to aIF2/eIF2 binding partners and the 40S ribosome.

**Fig. (4):**
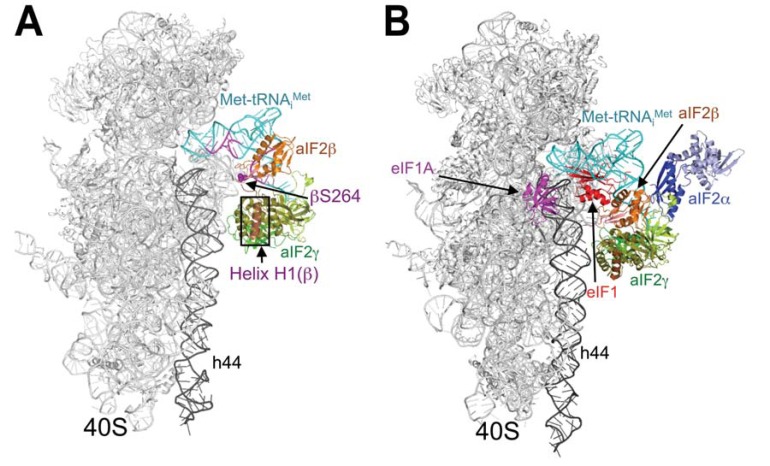
(**A**) Docking aIF2β on the 40S–aIF2γ–Met-tRNA_i_^Met^ complex model (adapted from [97]). Helix h1 of aIF2β, which forms the only rigid-body interaction with aIF2γ, is boxed. The aIF2β location corresponding to eIF2β-S264 is shown as purple spheres, and the Met-tRNA_i_^Met^ residues cleaved by Fe(II)-BABE linked to eIF2ΔC-βS264C are colored purple. (**B**) Docking of aIF2α, eIF1 and eIF1A on the 40S–aIF2βγ–Met-tRNA_i_^Met^ complex. Only the eIF1A core structure is shown leaving out both of its terminal tails.

The γ subunit of eIF2 shares considerable amino acid sequence and structural similarity with EF-Tu. Both aIF2γ and EF-Tu consist of three domains: an N-terminal GTP-binding domain and β-barrel domains II and III. Despite the structural similarity, it is anticipated that eIF2γ and EF-Tu will have different docking arrangements on the A- versus P-sites of the ribosome, and this might lead to differences in how the two factors bind aminoacyl-tRNAs [96]. Despite the extensive structural effort, the ribosome-contacting surfaces of aIF2 and eIF2 were until recently not known. The results of directed hydroxyl radical probing experiments now suggest that eIF2γ primarily contacts the acceptor stem of Met-tRNA_i_^Met^ and identify a key binding interface between domain III of eIF2γ and 18S rRNA helix h44 on the 40S subunit [97] (Fig. **4A - B**). Whereas the analogous domain III of EF-Tu contacts the T stem of tRNAs, biochemical analyses demonstrated that eIF2γ domain III is important for binding to ribosome and not to Met-tRNA_i_^Met^. On the other hand, mutations in the G domain or domain II of eIF2 were shown to alter the fidelity of AUG recognition most probably by affecting the conformation of initiator Met-tRNA_i_^Met^ binding to the P site [98,99].

eIF2β contains two domains conserved in all species: the N-terminal domain and the Zn-binding domain (ZBD) [92], as well as the eIF2γ-binding segment within the N-terminal domain [100]. This segment possesses three lysine repeats (K-boxes) that have been implicated in binding to the eIF2’s GAP in eIF5 and GEF in eIF2B, and nonspecifically also to RNA [25,101,102]. The N-terminal domain and the ZBD are connected by a relatively flexible helical region, however, no direct interactions between these two domains were detected [90,92]. Mutations altering conserved residues in the ZBD were demonstrated to increase initiation events at UUG codons [103]. These mutations seemed to allow eIF5-independent GTP hydrolysis and dissociation of Met-tRNA_i_^Met ^from eIF2•GTP *in vitro* suggesting that the ZBD inhibits GTP hydrolysis by eIF2γ. Indeed, according to the recent crystal structures of heterotrimeric aIF2 [89,95] it seems that it is the ZBD that makes the major contact with eIF2γ via its G domain. Even though there is a lot of flexibility in numerous structures of aIF2 that have been solved so far, which makes the predictions of where eIF2β sits on the 40S relatively speculative, the aforementioned hydroxyl radical probing study by Shin at el. indicated that it most probably occurs somewhere close to the A-site [97], where it could interact with other MFC components (see above and below for details) (Fig. **4A - B**). Interestingly, structural studies also revealed a similarity between the eIF2β-ZBD and the GAP domain of eIF5 occuring in its NTD [69]. Based on this it was hypothesized that the flexible, inhibitory eIF2β-ZBD might be displaced from the eIF2γ-G domain by the analogous ZBD in the eIF5-NTD as a means of stimulating GTP hydrolysis. In support, the eIF2γ-G domain was shown to directly interact with eIF5 *in vitro* [98].

The α subunit of eIF2 contains the critical Ser-51 residue that can be phosphorylated by several eIF2α-specific kinases and as such is essential for regulating the activity of eIF2 *in vivo *[104]. The three-dimensional structures of archaeal and human eIF2α proteins [88,93] indicate the presence of three distinct domains. The N-terminal domain I of aIF2α contains a β-barrel, with nonspecific RNA-binding activity (225), which was shown to interact with the central, helical domain II through a hydrophobic core. [105,106]. The C-terminal domain III then mediates the eIF2α interaction with the γ subunit via its domain II in the vicinity of the proposed binding pocket for methionine and A76 of initiator Met-tRNA_i_^Met^ [94]. Like the other two eIF2 subunits, eIF2α was also shown to promote AUG selection process. In reconstituted mammalian systems, mRNAs with thio-U substitutions at position -3 were cross-linked to eIF2α and RPS5 in the PICs, and the replacement of eIF2 with the eIF2βγ heterodimer reduced the efficiency of AUG recognition [107]. Based on these results it was hypothesized that eIF2α mediates a key contribution of the -3 base from the optimal Kozak consensus sequence [37] to the tight binding of Met-tRNA_i_^Met^ at the AUG codon and probably occurs near the E-site. Based on Shin *et al.*, however, the eIF2α seems to sit on top of eIF2γ and Met-tRNA_i_^Met ^beyond the P-site in the model PICs [97] (Fig. **4A - B**).

### eIF3 – the ORCHESTRA CONDUCTOR

**Fig. (5):**
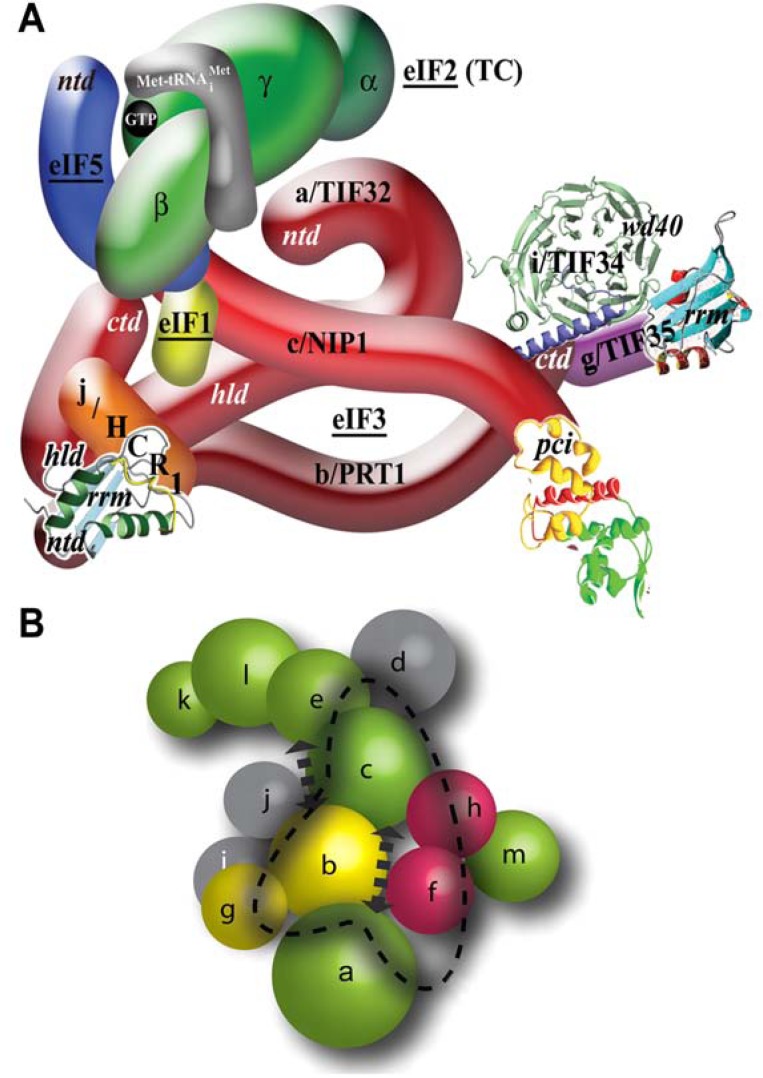
(**A**) A 3D model of eIF3 and its associated eIFs in the MFC (adapted from [38]). *ntd*, N-terminal domain; *ctd*, C-terminal domain; *hld*, HCR1-like domain; *rrm*, RNA recognition motif; *pci*, PCI domain; TC, ternary complex. The NMR structure of the interaction between the RRM of human eIF3b (green and light blue) and the N-terminal peptide of human eIF3j (yellow) [17], the NMR structure of the Cterminal RRM of human eIF3g (red and sky-blue) [18], the X-ray structure of the yeast i/TIF34 – b/PRT1-CTD complex [38], and the 3D homology model of the c/NIP1-CTD [59] were used to replace the original schematic representations of the corresponding molecules. (**B**) Proposed interaction model of human eIF3 based on tandem mass spectometry analysis (adapted from [109]). Subunit organization colored according to signature domains contained within the various subunits. PCI-containing domains (green), MPN domains (red), and RNA recognition motifs (yellow) show direct interactions with the exception of eIF3m and eIF3a. Subunits with no common signature domains are shown in gray. Dashed line is not relevant for the purpose of this review.

As indicated in the Introduction section, eIF3 has been demonstrated to promote or at least fine tune nearly every single step of translation initiation and now it seems that its influence reaches even beyond that. In budding yeast, eIF3 comprises five core essential subunits (a/TIF32, b/PRT1, c/NIP1, i/TIF34, and g/TIF35) and one non-core subunit (j/HCR1) (Fig. **5A**). These all have corresponding orthologs in the more complex mammalian eIF3, which contains seven additional non-conserved subunits (eIF3d, e, f, h, k, l, and m). Despite recent progress, the true composition of the core of mammalian eIF3 remains somewhat obscure. One study aimed at reconstitution of human eIF3 *in vitro* suggested that the functional core contains three non-conserved subunits e, f and h in place of eIF3i and g [108], whereas other work based on tandem mass spectrometry and solution disruption assays identified three stable modules, one of which, composed of a, b, i, and g subunits, closely resembled the yeast eIF3 core [109] (Fig. **5B**). The most recent work, based on co-expression and co-assembly of individual eIF3 subunits in *E. coli*, further added to this controversy by identifying the PCI/MPN octamer as the eIF3 core [110]. It is composed only of subunits containing the PCI (a, c, e, k, l, m) and MPN (f and h) domains; notably, the a and c subunits lack their C-terminal 1/2 and N-terminal 1/3 protein segments, respectively. Actually, the fact that the b subunit, generally considered as the eIF3 scaffold, did not stably co-purify with the PCI/MPN octamer could be caused by the absence of the CTD of eIF3a, which in its yeast homologue a/TIF32 carries the eIF3b/PRT1 binding site [111] (see below).

Whereas there is only very limited information on the subunit–subunit interaction web of mammalian eIF3, the labyrinth of mutual contacts among the yeast subunits has been mapped in great detail. As aforementioned, the b/PRT1 subunit serves as the major scaffolding subunit of eIF3 in both yeast and mammals making contacts with other core subunits (Fig. **5**) [12,109,112,113]. The b/PRT1 N-terminal domain contains a conserved RNA recognition motif (RRM) [17,114], which provides an interaction surface for the C-terminal half of a/TIF32 called the HCR1-like domain (HLD) and the NTD of j/HCR1 [17,111] (Fig. **5A**), followed by a middle domain predicted to fold into two β-propeller structures [115], the second of which contains a binding site for c/NIP1. Finally, the extreme CTD of the b/PRT1 scaffold forming the extended α-helix is required for association of i/TIF34 and g/TIF35 subunits [38,112]. Their binding is mutually co-operative as mutating the contacts between b/PRT1 and i/TIF34 not only prevents i/TIF34 association with eIF3 but also that of g/TIF35 [38]. i/TIF34 adopts a seven-bladed β-propeller structure made up of seven WD-40 repeats and interacts with the b/PRT1 via two contacts, one of which has an ionic and the other hydrophobic character (Fig. **5A**) [38]. g/TIF35 interacts with i/TIF34 and b/PRT1 through its NTD containing a predicted Zn-finger domain via yet to be defined binding sites [112]. The g/TIF35-CTD then adopts the RRM fold, whose NMR structure was resolved by RIKEN (Fig. **5A**) [18] and that is not involved in any subunit-subunit interactions [12,112]. The N-terminal domain of c/NIP1 makes direct contacts with eIFs 1 and 5, and *via *the latter also associates with the TC [12,116], serving as a critical nucleation center for the MFC formation. The following domain then interacts with the PCI domain of a/TIF32 and, towards the C-terminus, c/NIP1 captures the triangle-like network of interactions among all three large subunits by binding to b/PRT1. The actual CTD is formed by a canonical PCI domain (Fig. **5A**) [59]. Finally, a/TIF32 also contributes to the integrity of the MFC by contacting i) the TC via its extreme C-terminus and ii) eIF1 via its HLD [12]. Indeed, all aforementioned protein regions mediating the interactions of eIF3 subunits among themselves as well as with other components of the MFC are essential for cell viability and efficient translation.

**Fig. (6):**
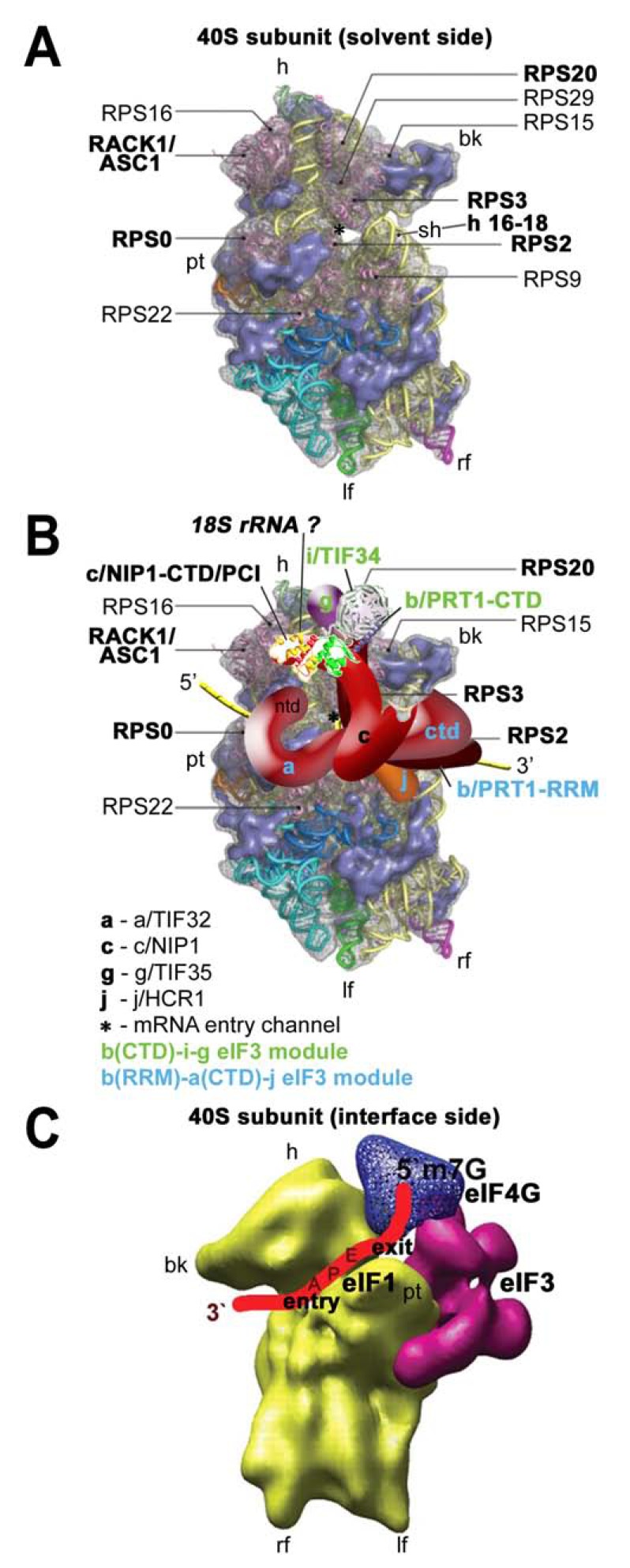
(**A – B**) A model of two eIF3 modules bound to the opposite termini of the scaffold b/PRT1 subunit situated near the mRNA entry channel of the 40S subunit. (A) The Cryo-EM reconstruction of the 40S subunit is shown from the solvent side with ribosomal RNA represented as tubes. Selected ribosomal proteins are shown as pink cartoons and labeled (adapted from [50]). Positions of RACK1/ASC1, RPS0, 2, 3 and 20 and 18S rRNA helices 16-18 are highlighted in bold. The mRNA entry channel is designated by an asterisk. (**B**) Hypothetical location of *S. cerevisiae* eIF3 on the back side of the 40S subunit based on the published interactions between RACK1/ASC1 and c/NIP1- CTD (and potentially also between c/NIP1-PCI and the 18S rRNA segments occurring in the head region) [59]; RPS0 and a/TIF32-NTD [41,117,118]; RPS2 and j/HCR1 [17]; RPS2 and 3 and a/TIF32-CTD [19]; helices 16-18 of 18S rRNA and a/TIF32-CTD [117]; and RPS3 and 20 and g/TIF35 [18] (see text for details). The schematic representations of b/PRT1-CTD and i/TIF34 and of the c/NIP1-CTD were replaced with the X-ray structure [38] or the 3D structural model [59], respectively, as in Fig. 5A. Two eIF3 modules represented by the b/PRT1-CTD–i/TIF34–g/TIF35 and the b/PRT1-RRM–a/TIF32-CTD–j/HCR1 are color-coded in green and blue, respectively. The yellow lines represent mRNA. (**C**) Cryo-EM docking model of mammalian 40S ribosome bound by eIF3 (violet), eIF4G (blue), eIF1 and mRNA. The A, P, and E sites and mRNA entry and exit sites are indicated; mRNA is depicted as a red line.

Systematic effort was devoted to mapping the binding site of eIF3 on the 40S. We found that the extreme NTD of a/TIF32 and the PCI domain in the c/NIP1-CTD form important intermolecular bridges between eIF3 and the 40S [41,59,117], and that the RRM of b/PRT1 similarly plays a direct role in anchoring eIF3 to the ribosome [14,17]. Unexpectedly, mutant eIF3 lacking i/TIF34 and g/TIF35 also showed reduced binding affinity towards 40S subunits *in vivo *suggesting that both small subunits further stabilize the core eIF3 in the PICs [38]. Finally, we observed that deleting the CTD of a/TIF32 reduced 40S association with the MFC when the connection between eIF3 and eIF5 (encoded by *TIF5*) in the MFC was impaired by the *tif5-7A *mutation [117]. Importantly, our findings that i) the C-terminal PCI domain of c/NIP1 interacts with the small ribosomal protein RACK1/ASC1/RPS33 (40S head) and probably also with 18S rRNA [59]; ii) that the a/TIF32-NTD binds to the RPS0A-CTT (mRNA exit channel) [117,118] and functionally interacts with mRNA sequences upstream of *GCN4* uORF1 that occur near the exit channel pore (see below) [41,42]; iii) that the a/TIF32-CTD contacts helices 16–18 of 18S rRNA [117] and RPS2 and RPS3 (all constituents of the mRNA entry channel) [19]; iv) that g/TIF35 binds the 40S beak proteins RPS3 and RPS20 (40S head) [18]; and that the j/HCR1-CTD interacts with RPS2 [17], all suggested that yeast eIF3 associates with the head and beak regions of the upper body of the solvent-exposed side of the 40S ribosome (Fig. **6A - B**). In support, deletion of ASC1 and conditional expression of RPS0A significantly impaired 40S-binding of eIF3 and all of its associated eIFs [59,118]. Interestingly, depletion of RPS5 (40S head) was previously also shown to affect 40S-binding of eIF3 [119], however, since it is not known whether and how RPS5 contacts eIF3, the molecular nature of this effect is unknown. Further consistent with these findings, in reconstituted mammalian 48S PICs, mRNA replaced with thio-U at positions -14 and -17 was cross-linked to eIF3a and eIF3d locating them at the mRNA exit channel; and hydroxyl radical cleavage mapping the mammalian eIF3 in the 48S PIC indicated that a segment of eIF3a interacts with helix 16 of 18S rRNA [120]. It should also be noted that besides RPS2, the j/HCR1-CTD also interacts with RPS23, situated near the ribosomal A-site on the 40S interface side [17]. Consistently, hydroxyl radical probing of the human eIF3j-CTD placed this domain in the 40S mRNA entry channel and in the ribosomal A-site [121]. Together these findings may suggest that either the j subunit protrudes the mRNA entry channel contacting both of its pores or it can bind to two different sites on the ribosome depending on its current role in the translational process.

Whereas the major eIF3 body sits on the 40S back, two domains of yeast eIF3 – the c/NIP1-NTD and the a/TIF32-CTD – were proposed to protrude under the beak area to the subunit interface reaching the ribosomal A-site (see summary model in Fig. **10**) [11,117]. Importantly, both of these interact either directly or indirectly with eIF1, the CTD of eIF5 [12,116], and *via* the β-subunit (encoded by *SUI3*) also with eIF2 (TC). As aforementioned, the binding site of eIF1 in the eIF1–40S complex lacking all other eIFs was mapped close to the ribosomal P-site in the interface platform area [52,64]. Hence, it is obvious that the eIF1 contact with the c/NIP1-NTD on the ribosome-bound MFC must be given up at a certain point of early initiation (perhaps during the switch from the close to open 40S conformations) for eIF1 to relocate to the ribosomal P-site area, as was posited earlier (Fig. **1**) [117]. The reciprocal move back to the c/NIP1-NTD could occur upon AUG selection, when eIF1 leaves the P-site to allow the Pi release, but the experimental evidence for this scenario is missing. Now, whereas it is not known where the eIF5-CTD resides, the very recent analysis indicating that eIF2β faces the ribosomal A-site (see above) [97] strongly suggests that the eIF5-CTD also occurs somewhere in this area (the eIF5-CTD is known to interact with the N-terminal K-boxes of eIF2β in the MFC [122]). These crucial findings thus could imply that the MFC-established c/NIP1-NTD–eIF5-CTD and a/TIF32-CTD–eIF2β contacts could be preserved also in the scanning PICs and provide eIF3 with direct means to actively regulate scanning as well as AUG recognition, as shown before (see also below) [11,14,17-19,38].

A somewhat similar 40S location was also proposed for mammalian eIF3, the low resolution (~30 A°) cryo-EM structure of which was recently resolved and revealed a complex possessing five domains that extend from a central body [110,123] (Fig. **6C**). Based on the low-resolution docking model of the eIF3–40S complex derived from cryo-EM reconstructions of two separate complexes between eIF3 and the hepatitis C virus IRES and between the IRES and the 40S subunit, it was proposed that the bulk of the eIF3 mass binds to the back side of the 40S subunit [123]; however, the major mass was shifted towards the platform and not the beak as in the yeast model. Only the future structural experiments will tell whether this difference simply reflects specific differences between lower and higher eukaryotes or whether the yeast binding map or the mammalian docking model is incorrect; it is also conceivable that the position of eIF3 in the canonical 43-48S PICs differs significantly from the one that eIF3 adopts in the pre-initiation complexes hijacked by viral IRESes.

As for the canonical functions of the individual subunits, several important segments (designated as Boxes) within the c/NIP1-NTD were identified, mutations of which impaired the TC recruitment to the 40S ribosomes and relaxed stringency of the start codon selection producing the so called Sui^- ^phenotype ([11] and M. Karaskova and L.S.V., unpublished observations). Mutants imparting the Sui^-^ phenotype (suppressor of initiation codon mutation) allow increased utilization of near-cognate codons (UUG or AUU). Similarly, j/HCR1, as the only non-essential subunit of yeast eIF3, was shown to form together with the a/TIF32-CTD and the RRM of b/PRT1 an eIF3 subassembly that ensures stringency of the AUG start codon selection by blocking leaky scanning (skipping AUGs) [14,17,19]. In support of this, the former two subunits interact with the components of the 40S open/closed-state-switching latch, as mentioned above. A robust leaky scanning phenotype was also observed with mutations disrupting the web of interactions among the b/PRT1-CTD and i/TIF34 and g/TIF35, located most likely above the mRNA entry channel. Hence it appears now that the scaffold b/PRT1 subunit serves to connect two eIF3 modules at each of its termini (a/TIF32-CTD–j/HCR1 at the N-terminal RRM, and i/TIF34–g/TIF35 at the C-terminal α-helix) that work together and with c/NIP1 [11] and other eIFs [32] to fine-tune the AUG selection process (Fig. **6B**). The a/TIF32-HLD seems to play an important role in the critical activity of yeast eIF3 in productive mRNA recruitment [15,19,20]. The fact that several eIF3 subunits are known to directly interact with mRNA [18,41,59,120,124,125] furthermore suggests that the way the mRNA interacts with the mRNA binding channel during scanning for AUG can be influenced by eIF3. Consistently, the very original *prt1-1 *point mutation in b/PRT1, single-point substitutions in the conserved KERR motif of the a/TIF32-HLD as well as mutations in i/TIF34 and the g/TIF35-RRM were shown to affect either the rate or processivity of ribosomal scanning [13,19].

There is very limited functional information on canonical roles of mammalian eIF3 with the exception of a handful of studies. *In vitro* experiments revealed that human eIF3j can bind to the 40S subunit by itself and is required for stable 40S-association of purified eIF3 [67,113,126]. In the absence of other factors, eIF3j was also demonstrated to be mutually antagonistic for binding to the 40S subunit with mRNA and with eIF1A [67,121]. These results together with the aforementioned determination of the position of the eIF3j-CTD in the 40S mRNA entry channel and the ribosomal A site by hydroxyl radical probing [121] led the authors to suggest that eIF3j may coordinate binding of mRNA and eIFs within the decoding center. However, given the fact that deletion of its yeast homologue is not lethal [127], the true physiological importance of these observations awaits careful examination in living mammalian cells. In the other pioneering study that worked with reconstituted mammalian eIF3 it was suggested that eIF3i and g, the yeast homologues of which are essential [128], are dispensable for formation of the active 48S PIC *in vitro* [108].

Besides playing these canonical roles in general translation initiation, eIF3 was also implicated in regulation of protein synthesis during viral infections [129], in mRNA surveillance by the nonsense-mediated decay pathway (NMD) [130], in signal transduction pathways by recruiting protein kinases such as mTORC1 and S6K to the surface of the 40S subunit [131,132], and in the gene-specific translational control mechanism termed reinitiation (REI) in yeast, plant and mammalian cells [41,42,133,134], which is discussed in detail below.

### eIF3 – the TRANSLATIONAL CONTROL-ACTOR

There are a few examples in eukaryotes that deviate from the general translation initiation mechanism described in the Introduction section and start protein synthesis either without scanning or at internal sites of an mRNA. These involve initiation promoted via internal ribosomal entry sites (IRES) [135], ribosomal shunting [136], or reinitiation (REI) after translation of either short or long upstream open reading frames (uORFs) [137-140]. Many of these mechanisms are utilized by invading viruses or serve as the regulatory means for gene-specific translational control of mostly regulatory proteins such as transcription factors and proto-oncogenes [141]. 

Short uORFs are present in approximately 13% of yeast and 50% of human transcripts [10] suggesting that they represent a comprehensive cis-regulatory function in translational control of eukaryotic gene expression. The presence of a short uORF in mRNA’s 5‘ UTR generally leads to significant reduction in expression of a downstream major ORF, the degree of which clearly depends on the “strength” of the Kozak nucleotide context surrounding the initiating AUG of a given short uORF. There are several ways how short uORFs regulate gene expression in a gene-specific manner (reviewed in [138]) but for the purpose of this review I will focus only on those uORFs that allow relatively efficient REI after their own translation.

For an uORF to allow efficient REI downstream, ribosomes initiate in the normal way at its AUG (or near cognate) start codon; however, at the termination codon, only the 60S but not the 40S subunit dissociates from mRNA in an incomplete ribosomal recycling reaction. Thus retained 40S subunit subsequently resumes scanning downstream and recruits the Met-tRNA_i_^Met ^(in a form of a ternary complex with eIF2•GTP) along the way to be able to “re-initiate” again at a next AUG start site. It has been shown that the ability of some uORFs to retain the 40S subunit on the same mRNA molecule after it has terminated translation at the uORF’s stop codon depends on: (i) *cis*–acting mRNA features surrounding a given uORF, (ii) the time required for the uORF translation, which is determined by the relative length of a short uORF and the translation elongation rates, and, finally, (iii) on various initiation factors (reviewed in [137,138,141]). The last two requirements are neatly united in the long-standing idea that eIFs important for promoting reinitiation remain at least transiently associated with the elongating ribosome, and that increasing the uORF length or the ribosome transit time increases the likelihood that these factors are dropped off [142,143]. We recently provided genetic evidence for this hypothesis showing that in yeast *S. cerevisiae,* eIF3 remains 80S-bound for several rounds of elongation and critically enhances the REI capacity of post-termination 40S ribosomes [41]. Among other eIFs that have been implicated in promoting efficient REI up to date are mammalian eIF4A and eIF4G [43]; however, their functional contributions to REI remain to be elucidated. With respect to *cis-*acting features, with the exception of the uORF-mediated translational control of the budding yeast *GCN4* described below, there is virtually nothing known about what other REI-promoting mRNA features are required. Finally, REI efficiency is also directly dependent on (iv) the distance between the uORF termination codon and a downstream initiation codon owing to the fact that the rescanning PICs require a certain time for *de novo* recruitment of the TC to be able to decode the next AUG start site [104]. Therefore, REI can be delicately regulated by manipulating the TC levels via eIF2α-specific protein kinases such as GCN2. 

**Fig. (7):**
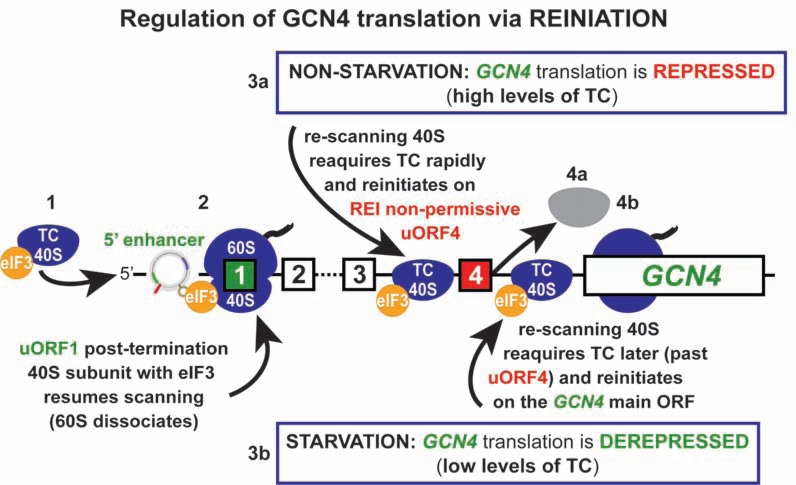
(**A**) Schematic of the *GCN4* mRNA leader showing distribution of all four short uORFs (REI-permissive uORF1 is labeled green; REI-non-permissive uORF4 is labeled red), the predicted structure of the uORF1’s 5’ *cis*-acting sequences (5’ enhancer), 40S- and 80S-bound eIF3, and the description of the mechanism of the *GCN4* translation control (adapted from [42]). The 3a and 4a “*GCN4*-expression repressed” steps take places under non-starvation conditions with abundant TC levels, whereas the 3b and 4b “*GCN4*-expression derepressed” steps occur under starvation condition with limited supply of the TC (see text for further details).

Translation of *GCN4*, transcriptional activator of numerous biosynthetic genes is regulated mainly in response to amino acids availability and relies on the presence of four upstream open reading frames (uORFs 1 – 4) in its mRNA leader (Fig. **7**) (reviewed in [137]). After translation of the first REI-permissive uORF1, a sizeable number of small ribosomal subunits does not dissociate from the *GCN4 *mRNA and instead resumes scanning downstream. In order to reinitiate on any of the downstream uORFs or on the main *GCN4 *ORF, these re-scanning subunits have to first reacquire the TC. When cells are grown in rich media, intracellular levels of the TC are high, so most of re-scanning ribosomes pick it up before reaching the AUG start codon of the inhibitory (REI-nonpermissive) uORF4, at which they reinitiate. This uORF does not allow resumption of scanning of post-termination 40S ribosomes and thus blocks further reinitiation. However, when cells are starved for amino acids, the GCN2 kinase induces dramatic decrease in the TC levels, which enables many of the re-scanning ribosomes to skip the trap of uORF4 by picking up the TC after scanning through it. As a result, the AUG of *GCN4* is reached and its ORF gets translated even though the global protein synthesis is significantly down-regulated.

The long-standing paradox why ribosomes readily reinitiate after translation of uORF1 but not uORF4 has been partially resolved only recently. The original analyses proposed that AU-rich *cis*-acting sequences surrounding the stop codon of uORF1 might favor resumption of scanning and REI, whereas GC-rich sequences flanking the uORF4 stop codon could trigger ribosome release [144]. However, it seems that this cannot possibly be the complete explanation, because uORFs-2 and-3 also have AU rich sequences downstream of the stop codon, yet they have been considered as REI-nonpermissive uORFs so far, like uORF4 [137]. In addition, sequences 5’ of uORF1 were also shown to be critical for efficient REI. In contrast to the 3’ sequences, the true molecular mechanism of which remains to be explored, the molecular contribution of the *cis*-acting 5’ sequences has been recently elucidated [41]*.* It was demonstrated that the 5’ enhancer of uORF1 functionally interacts with the extreme NTD of the a/TIF32 subunit of eIF3 and that establishment of this contact post-termination is crucial for stabilization of the small ribosomal subunit on the mRNA. This step then greatly facilitates efficient resumption of scanning of the 40S ribosome for reinitiation downstream. In support of this, the immediately following region still within the NTD of a/TIF32 interacts with the small ribosomal protein RPS0 [118], as aforementioned, which is positioned near the mRNA exit pore on the solvent side of the small subunit, where the uORF1’s 5’ enhancer occurs on the post-termination 40S ribosome. Four particularly critical nucleotide sequence and/or structural motifs called REI-promoting elements (RPEs) have been delineated, two of which were shown to operate in the a/TIF32-NTD dependent manner [42]. Similarly, alanine substitutions of consecutive blocks of 10 residues throughout the a/TIF32-NTD revealed three particularly critical REI motifs (amino acids 51-60, 71-80 and 161-170) [42]. Together these findings led to a model (Fig. **7**) in which wild-type eIF3 remains at least transiently associated with the translating 80S ribosome via RPS0 and other mutual contacts with the solvent-exposed side of the 40S subunit, and if it does not drop off prior to termination, the extreme NTD of a/TIF32 interacts with the 5’ enhancer to permit ribosomal recycling of only the large 60S subunit, while aiding to preserve the small subunit on the *GCN4 *mRNA [41,42]. Besides the *GCN4 *mRNA leader, there is another well described example of a REI-permissive uORF in yeast represented by uORF of the *YAP1 *gene, an AP1-like transcription factor [145]. The most recent insights showing that the *YAP1* reinitiation mechanism also operates in the a/TIF32-NTD-dependent manner and that the 5’ sequences of its uORF contain 5´-enhancer motifs similar to *GCN4*‘s uORF1 strongly suggests that the underlying mechanism of reinitiation on short uORFs is conserved, at least in yeast [42].

A related but not identical mechanism has been shown to govern expression of the mammalian functional homologue of *GCN4*, the* ATF4 *transcription factor, and also that of the bZIP transcriptional regulator *ATF5* (both of which were implicated in mediating the unfolded protein stress response [146,147]), indicating that at least basic principles of this regulatory system have been evolutionary conserved. Central to the *ATF4* translational control is also the 5'-leader of the *ATF4* mRNA that encodes two uORFs with different characteristics. The first *ATF4* uORF encodes a polypeptide only 3 amino acid residues in length, whereas the second uORF is 59 amino acid residues in length and overlaps the first 83 nt of the *ATF4*-coding region. The *ATF4* translation begins with the 48S PIC scanning from the 5'-end of the *ATF4* mRNA and initiating translation at the REI-permissive uORF1 [146]. Following uORF1 translation, the 40S subunit retains association with *ATF4* mRNA and resumes scanning downstream. In non-stressed cells, when eIF2α phosphorylation is low and the amount of the TC is high, re-scanning ribosomes readily reinitiate translation at the next extended uORF2. Following translation of uORF2, ribosomes terminating already in the *ATF4* coding region dissociate from the transcript and thus prevent translation of *ATF4*. During stress conditions, elevated phosphorylation of eIF2α reduces the TC levels, thus increasing the time required for re-scanning ribosomes to become competent to reinitiate again. Hence following uORF1 translation, delayed reinitiation allows a portion of the re-scanning 40S ribosomes to bypass the uORF2 initiation codon, and translate *ATF4* instead [146]. Despite these obvious similarities, it is not known whether or not the 5’ and 3’ sequences flanking uORF1 of *ATF4* (and in fact of any short uORF in higher eukaryotes in general) and eIF3 have the same importance for REI in mammals as they do in yeast. In other words, the molecular details of this *GCN4*-related mechanism in mammals only await their elucidation.

The reinitiation mechanism mediated by short uORFs bears a significant resemblance to the RNA-viruses specific termination/reinitiation mechanism that is the best described for the polycistronic mRNA of feline calicivirus [134,139,148]. A specific 87-nt element (called TURBS) preceding the overlapping termination/initiation site of two long ORFs 2 and 3 folds into a special secondary structure that not only presents its motif 1 for base-pairing with a specific segment of 18S rRNA, but also interacts with eIF3 via several subunits including eIF3a and eIF3g. This net of interactions is believed to prevent dissociation of the mRNA/eIF3/40S complex in order to allow efficient REI on ORF3. Interestingly, the proposed role of eIF3 in the latter mechanism may hypothetically indicate that eIF3 could contribute to efficient REI in mammalian cells also on short uORFs. 

### eIF4F & eIF4B – the mRNA DELIVERYMAN & the MOLECULAR ‘COAT RACK’

eIF4F forms a thermodynamically stable complex composed of three proteins: the cap binding eIF4E, the scaffold eIF4G, and the DEAD-box helicase eIF4A. eIF4E binds to the 7-methylguanosine cap structure of mRNA and to eIF4G that together with eIF4A enhances the rate of mRNA recruitment to the 43S PIC producing the 48S PIC (43S–eIF4F– mRNA), which they further stabilize. The presence of eIF4A in eIF4F strongly suggests that it is the helicase that generates a single-stranded binding site – the “landing pad” – for the 43S PIC at the very 5’ end of structured mRNAs. eIF4A bound to eIF4G is also required to remove all secondary structures that may occur downstream the 5’ cap to allow the mRNA to pass through the 40S mRNA entry or exit channels and permit selection of the AUG start site in the P site (reviewed in [10]). Critical evidence that eIF4F and ATP hydrolysis by eIF4A stimulate scanning through the secondary structures was elegantly provided by the Pestova’s group in the mammalian reconstituted system. They showed that the 48S PIC assembly on a synthetic mRNA lacking any secondary structures did not require eIF4F [31]; however, presence of a stem-loop imposed a strong requirement for the eIF4 factors and ATP.

eIF4G serves as a molecular scaffold that can be divided into three sections: the NTD containing binding sites for poly(A)-binding protein (PABP) and for RNA; the central core nesting binding sites for eIF4E, eIF4A (the first site of two formed by the HEAT-repeat domain), eIF3, mRNA (two sites); and finally the CTD carrying the second binding site for eIF4A and a site for the MAP kinase-interacting kinase 1 (Mnk1) [149-154]. Interestingly, yeast eIF4G contains additional RNA binding site in place of the eIF3 binding site and lacks the entire CTD (see below). A study addressing the roles of individual binding activities of mammalian eIF4G somewhat unexpectedly revealed that the loss of any one of its interactions had a minimal effect on the ability of eIF4GI to support initiation of translation in cells [26]. In contrast, in the reticulocyte lysates proteolytically depleted of endogenous eIF4G, mutant eIF4G variants unable to interact with eIF4A or eIF3 (but not with PABP) markedly decreased its ability to support translation, possibly due to the fact that they had to compete with eIF4G proteolytic degradation products. In any case the authors proposed that there is considerable redundancy in the mechanisms forming 48S PICs in mammalian cells, such that many individual interactions have regulatory rather than essential roles (see also below) [26]. 

Despite the fact that a high-resolution structure of the entire eIF4G protein has not been possible to obtain so far, individual functional domains have been solved such as the PABP-binding domain [155], the eIF4E-binding domain [156], and the two eIF4A-binding domains [157,158]. The tight binding of eIF4F to the capped 5’ end is dependent not only on the eIF4E-cap interaction and eIF4E binding to eIF4G, but also on the RNA-binding activity of eIF4G and the direct interaction of eIF4G with PABP bound to the poly(A) tail [159-163]. eIF4G has also been suggested to work as the molecular bridge stimulating mRNA attachment to the 43S PIC by interacting simultaneously with mRNA and factors bound the PIC, including eIF3 in mammals (via the eIF3e subunit [24]) and eIF5 and eIF1 in yeast (both of the latter eIFs were shown to bind to the NTD of c/NIP1 subunit of eIF3 simultaneously with eIF4G; hence even though eIF3 and eIF4G do not interact directly in yeast, their interaction could be bridged [25]). However, the fact that mutating the eIF3-binding site in mammalian eIF4G had no impact on initiation in cells, as mentioned above, and that this mutant form of eIF4GI remained associated with polysomes in siRNA-treated HeLa cells together suggested that the eIF4G–eIF3 interaction may not be essential for association of eIF4F and mRNA with the 43S PICs in mammalian cells and thus could be dispensable for basal translation in general [26]. By the same token, depletion of eIF4G from yeast cells did not provoke a significant decrease in the amount of two short “native reporter mRNAs” encoding *RPL41A* and *MFA2* associated with PICs *in vivo*, although the depletion of eIF2 or eIF3 did [15]. Furthermore, omission of eIF4G from the yeast reconstituted system only reduced the kinetics of mRNA recruitment but had no impact on the overall endpoints of 48S PIC assembly for two model mRNAs, whereas omission of eIF3 produced no complexes whatsoever [20]. Together these results suggested that yeast eIF4G significantly stimulates the rate of mRNA attachment to the PICs *in vivo *but is also not essential for basal level of translation of most mRNAs. Indeed, two independent studies showed that changing the eIF4G levels in yeast cells did not dramatically impair translation of any particular mRNAs but rather had a differential impact on their translational efficiencies [28,29]. Consistently, Ramirez-Valle *et al.* depleted eIF4GI in mammalian cells and also observed merely mRNA-specific effects on translational efficiency, but no robust reduction in overall protein synthesis [27]. That leaves us with an open question of what is the canonical role of eIF4G – and its interaction with eIF3 in mammals – in general translation initiation? Does it promote the onset of scanning rather than the mRNA recruitment *per se*? Lorsch *et al.* in fact suggested that the effect of eIF4G on the observed rate of mRNA recruitment *in vitro* could come, at least in part, from a role in increasing the efficiency or processivity of scanning [20]. The aforementioned data from the reconstituted mammalian system showing that eIF4F is not required for mRNAs harboring unstructured 5’ UTRs to be attached to the 43S PICs and scanned for the AUG start codon, but becomes indispensable for locating the start on mRNAs with structured 5’ UTRs [31] also seems consistent with the latter possibility. As for the eIF4G–eIF3 interaction, it was recently reported that the affinity of eIF4G for eIF3 is regulated by insulin signaling through the mTORC1 complex [131,132]. Hence it seems plausible that the eIF4G–eIF3 contact may play a critical role in regulating cell proliferation by altering the expression levels of mRNAs in response to cell signaling events in a gene-specific manner.

eIF4E resembles a cupped hand with a curved β-sheet consisting of eight antiparallel strands, supported by three additional α-helices [164,165]. The cap structure binds in the concave surface, sandwiched by two conserved tryptophan residues with a third tryptophan helping to stabilize the complex. eIF4E binds eIF4G via its NTD and this interaction supposedly induces a folding transition in both eIF4E and eIF4G, perhaps explaining the enhanced affinity of eIF4E for the cap structure in the presence of eIF4G [156,166-169]. There are many eIF4E family members that appear to have different roles in different tissues providing the cell with a powerful mechanism of translational control in cell growth and development (reviewed in [170]). Besides eIF4G, eIF4E binds to a number of proteins called eIF4E-binding proteins (4E-BPs), which possess similar binding determinants as the eIF4E-binding site in eIF4G implying that they interact with the same domain in eIF4E [171]. These proteins, when hypo-phosphorylated, compete with eIF4G for binding to eIF4E as the means for the second major principle of general translational control responding to various environmental changes (Fig. **1**) (reviewed in [1]). The stimulation of cell growth activates various signaling pathways that ultimately phosphorylate 4E-BPs on multiple sites to lower their affinity to eIF4E in order to allow formation of the activated eIF4F complex [172,173]. 

eIF4A is a typical DEAD-box helicase protein that possesses two RecA-like domains widely separated in a fully open conformation by a flexible linker [174]. It exhibits RNA-dependent ATPase activity and ATP-dependent duplex unwinding activity that are both activated by association of eIF4A with eIF4G (reviewed in [175]). It is thought that the latter activity arise from the ability of both RecA-like domains to bind and hydrolyze ATP making eIF4A to alternate between open and closed conformations, which powers the unwinding. Interestingly, the energy of ATP hydrolysis is not required to produce single-stranded regions by the strand separation *per se*, rather it appears that ATP hydrolysis serves primarily to dissociate the enzyme from the RNA, recycling it for multiple rounds of RNA binding and melting [176]. Not surprisingly, it was shown that the interaction of eIF4A with the eIF4G HEAT-repeat domain in both yeast and mammals increases eIF4A’s ability to stimulate translation initiation both *in vitro *and *in vivo* [151,177-180]. Besides its incorporation into the eIF4F complex, the helicase activity of eIF4A is also stimulated by eIF4B (see below) and eIF4H [181-184]. The crystal structure of a complex between eIF4A and the eIF4G HEAT domain suggested that eIF4G functions as a “soft clamp” that stabilizes the active/closed conformation of eIF4A by interacting with both the NTD and the CTD of eIF4A via C- and N-terminal α-helices, respectively, in the HEAT domain [185,186].

eIF4B and eIF4H are two accessory proteins shown to weakly interact with eIF4A [187-190] and to stimulate its helicase activity by increasing eIF4A’s affinity for RNA and ATP and also by holding on to the single-stranded regions created by eIF4A – hence serving as some sort of the „molecular coat racks“ (a quote from Jon Lorsch) [182,191]. Indeed, both eIF4B and eIF4H, which share sequence similarity across the length of eIF4H, were experimentally demonstrated to possess RNA binding activity through a conserved N-terminal RNA-recognition motif (RRM) that, at least in the case of yeast eIF4B, plays an important role in RNA strand-exchange activity [192]. Considering that both of these eIFs stabilize the closed (ATP-bound) conformation of eIF4A [188], it was hypothesized that eIF4B and eIF4H mechanistically stimulate eIF4A helicase activity by enhancing domain closure in the manner described above for eIF4G [189]. In fact, the accessory/regulatory character of eIF4B is nicely illustrated by the fact that the deletion of eIF4B (encoded by *TIF3*) from yeast (there is no yeast homologue of eIF4H) is not essential and instead results “only” in a severe slow-growth phenotype [193,194].

poly(A)-binding protein (PABP) was demonstrated to be a *bona fide* translation initiation factor in mammalian *in vitro* translation extracts [195]. It interacts with eIF4G via its NTD and this interaction is expected to represent one of the ways promoting mRNA circularization – connecting the cap and the poly(A) tail in a circle [149,163,196]. However, the recent data shed some doubts over the physiological importance of the “mRNA closed-loop” specifically generated through the interaction between PABP and eIF4G, at least in yeast, as it was shown that the PABP-eIF4G interaction is dispensable for wt cell growth and becomes important for viability only when the eIF4E-cap interaction and an RNA-binding domain in the eIF4G-NTD are simultaneously impaired [160]. Hence it was proposed that the eIF4G-PABP interaction is not critical for efficient initiation, but simply represents one of several interactions that stabilize eIF4G binding to mRNA. In support of this proposal, Hinton *et al.* did not detect any significant effect on the ability of mammalian eIF4GI deficient for binding to PABP to support translation in cells as well as in reticulocyte lysates, as noted above [26]. It is thus entirely possible that the PABP’s stimulatory role in translation initiation might involve an interaction(s) with other factor(s) than eIF4G, as suggested earlier [15,197-199], some of which may promote formation of the “mRNA closed-loop” independent of eIF4G [198].

**Fig. (8):**
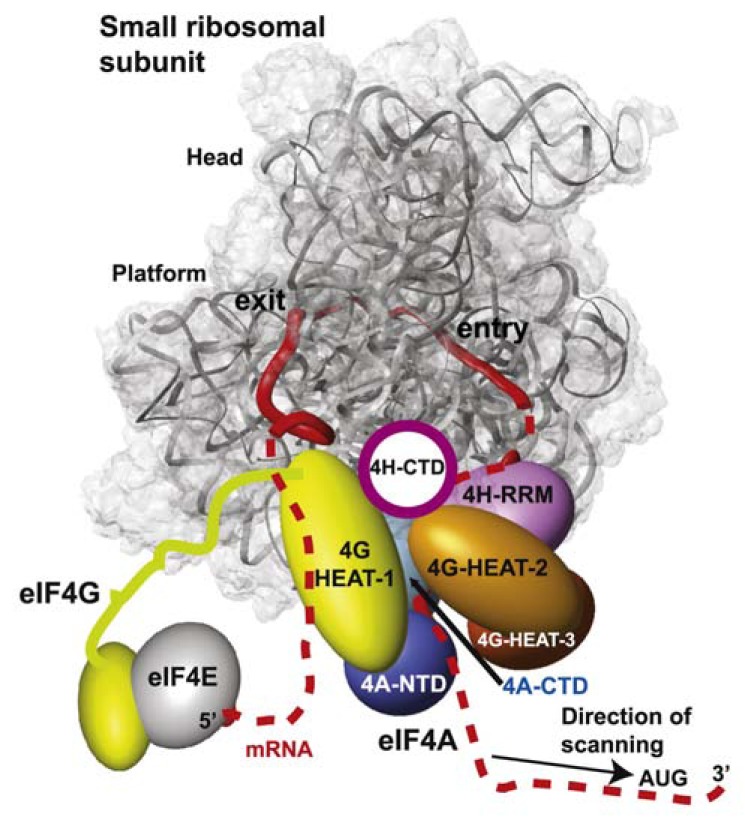
Hypothetical model for the mechanism of unwinding of mRNA and scanning by the 48S PIC. The small ribosomal subunit is viewed from the solvent face in a gray semi-transparent surface with the rRNA backbone shown as ribbon; the mRNA is illustrated as red solid ribbon. Schematic shows the PIC-associated factors and/or their particular domains including eIF4E and the 4E-BR, the HEAT1, HEAT2 and HEAT3 domains of eIF4G, the CTD and RRM domains of eIF4H, and the NTD and CTD of eIF4A. The direction of scanning of the initiation complex along the mRNA (5' to 3') is indicated by an arrow; eIF4A is shown on the leading (3') side of the scanning complex.

Despite an extensive effort by many groups, there are relatively major discrepancies in the predictions where the eIF4F complex is situated within the scanning PIC. The integration of two separate cryo-EM docking models of the mammalian eIF3-40S complex (already based on cryo-EM reconstructions of two separate complexes between eIF3 and the hepatitis C virus IRES and between the IRES and the 40S subunit – see above) and of the eIF3-eIF4G assembly led to the prediction that eIF4G interacts with the 40S head region, above the platform and the mRNA exit channel, which would position eIF4F upstream of the scanning PIC. The authors proposed that this way, eIF4F would prevent the 3’ to 5’ backsliding of the PIC rather than the unwinding of the secondary structures in front of the ribosome [123]. A different model suggests that eIF4G can span the mRNA exit and entry channels on the 40S subunit and places eIF4A at the entry channel for the unwinding of the structures ahead of the ribosome. According to this model, mRNA emerging from the exit channel would be additionally bound by the eIF4G HEAT-1 domain (Fig. **8**). eIF4B or 4H are predicted to occur behind eIF4A but in front of the entry pore to hold on to the single-stranded regions of the unwound mRNA. If true, it would imply that the 48S PIC remains close to the cap even during scanning and the already-inspected nucleotides form a loop between the cap and the traversing ribosome [189]. Finally, hydroxyl radical cleavages directed from eIF4G HEAT-1 in reconstituted mammalian 48S PICs occurred primarily in expansion segment 6 (ES6) of 18S rRNA, which emerges at the solvent side of the platform just below helix 26 and branches into three long irregular helices. These results suggest that at least the HEAT-1 domain is situated below the platform, close to the “left foot” of the 40S subunit [200], which is not entirely consistent with the predicted position for this domain near the exit channel by the second model. Whichever model is correct, there seems to be at least one thing for certain and that is that the eIF4F complex binds to the back – solvent exposed side of the ribosome, similar to another multiprotein complex – eIF3.

### eIF5 – the EXECUTOR

eIF5 can be divided into two functionally and structurally different halves. The N-terminal half acts as the GAP (GTP-ase accelerating protein) for eIF2 [201-203] and contains a domain similar to the C-terminal Zn-binding domain (ZBD) in eIF2β [69]. It also harbors an “Arg finger” in Arg15 as the critical residue for its GAP activity, which is thought to be pushed into the GTP-binding pocket of eIF2γ to stabilize the transition state for GTP hydrolysis [33,203,204]. That pretty much defines where at least the NTD of eIF5 occurs on the ribosome – next to eIF2γ, with which it interacts [98], even though a precise ribosomal location of eIF5 has not been experimentally determined as yet. Interestingly, structural similarity between eIF1 and the eIF5-NTD was also observed [62,69], which raised the possibility that eIF5 and eIF1 might compete for binding to the 40S platform. It was even proposed that eIF1 dissociation or displacement upon AUG recognition would clear the way for the eIF5-NTD to dissociate from eIF2 and bind in place of eIF1 on the 40S platform to actually allow the Pi release and stabilize the closed conformation [68]. Obviously, structural work is very much needed to prove or disprove these intriguing yet speculative ideas. The CTD is an important nucleation center for the yeast MFC making direct contacts with eIF1, the c/NIP1-NTD and the eIF2β-NTD [16,25,101,116,205]. It possesses a HEAT repeat domain [206,207] that can be also found in the catalytic subunit of eIF2Bε [208] and eIF4G [157]. As hinted above, based on the recent analysis of the eIF2 whereabouts on the ribosome indicating that eIF2β faces the A-site in the PIC [97], there is a reason to assume that the CTD of eIF5 also occurs somewhere in this area, where it could interact with the NTD and CTD of c/NIP1 or a/TIF32, respectively [12,117]. Apart from the GAP role, eIF5 was also demonstrated to exert several non-GAP functions. As mentioned above, it is thought to stimulate eIF1 displacement from the P-site upon AUG recognition [68], and by binding to eIF1A to prevent rebinding of eIF1 to the platform in order to stabilize the closed/scanning arrested conformation [34]. Towards the end of its translational cycle life, eIF5 is ejected from the ribosome together with eIF2•GDP. Actually, it was recently shown that eIF5 actively stabilizes this “discharged” form of eIF2 preventing the GEF reaction by eIF2B [46,47]. Therefore it was suggested that eIF5 should be considered as a bi-functional protein that acts upon eIF2 as a GDP dissociation inhibitor (GDI) as well as a GTP-ase accelerator (GAP).

In analogy with the autoregulatory mode of eIF1 expression mentioned above [72], eIF5 was very recently also found to be a subject of an autoregulatory mechanism of its own expression [209]. Many eukaryotic mRNAs encoding eIF5 seem to contain one or more upstream open reading frames (uORFs), whose start codons are in poor contexts. Overexpression of eIF5 was demonstrated to increase initiation at these “poor-context” uORF and thus to inhibit its own synthesis. Hence overexpression of eIF5 or eIF1 confers opposing effects on stringency of AUG selection. eIF1 overexpression increases the stringency of start codon selection resulting in reduced initiation at its own “poor-context” AUG and at the “poor-context” AUGs of eIF5’s uORFs (and presumably the “poor-context” AUGs in general) – as a consequence, eIF1 translation decreases and eIF5 translation increases. eIF5 overexpression relaxes the stringency of start codon selection resulting in increased initiation at eIF1’s AUG and at AUGs of eIF5’s uORFs (and presumably the “poor-context” AUGs in general) – as a consequence, eIF1 translation increases and eIF5 translation decreases. Overexpression of both factors at the same time practically canceled out each other’s effect. These findings thus strongly argue that the mode of expression of eIF1 and eIF5 establishes a higher-order cross-regulatory mechanism that fine tunes the stringency of start codon selection.

### eIF5B – the FINAL TRIGGER

The initiation phase is completed by the subunit joining step producing an 80S initiation complex (IC) poised for elongation. Since majority of the initiation factors occupy the subunit interface, joining of the 60S subunit leads to ejection of most of them with the exception of eIF1A (see below; eIF3 and eIF4F, which are situated on the solvent-exposed side of the ribosome, were proposed to stay 80S-bound as well as mentioned above) [41-43,67,210]. This closing step is promoted by eIF5B [39,211], a ribosome-dependent GTPase that is homologous to prokaryotic initiation factor IF2 [212]. However, whereas bacterial IF2 is responsible for selection of fMet-tRNA_f_^Met ^and stabilization of its binding to the 30S ribosomal subunit, no binding of eIF5B with Met-tRNA_i_^Met^ has been reported in eukaryotes. Nevertheless, a recent report by Terenin *et al.* showed that, under stress conditions inactivating eIF2 by phosphorylation, the hepatitis C virus (HCV) internal ribosome entry site (IRES) can drive translation with eIF5B and eIF3 as the only initiation factors [213]. This suggests that eIF5B can in fact interact with and stabilize Met-tRNA_i_^Met^ directly on the ribosome, similar to IF2. 

**Fig. (9):**
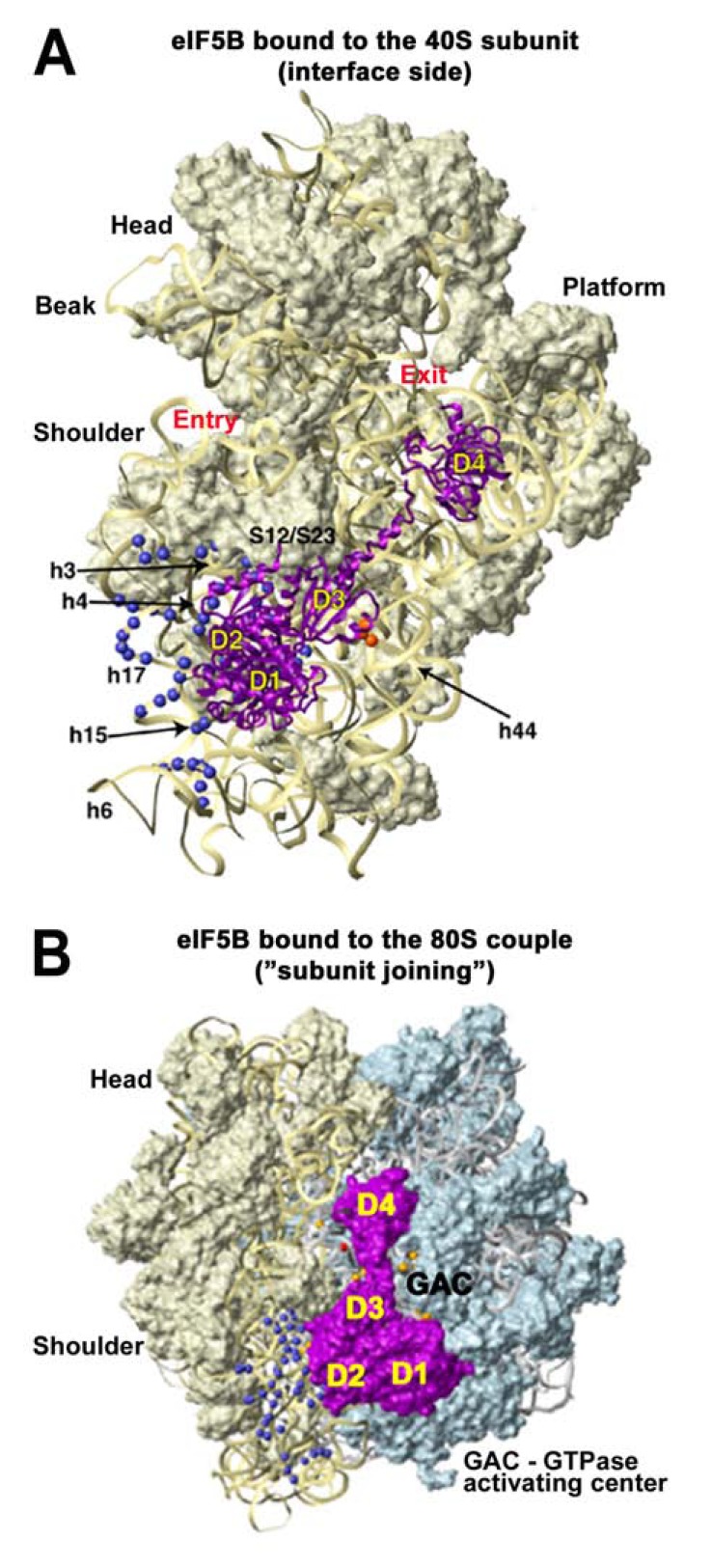
(**A**) The modeled position of eIF5B (purple ribbon) relative to the 30S subunit in the *E. coli* 70S ribosome crystal structure (adapted from [75]). Blue and orange spheres represent hydroxylradical cleavage positions in 18S rRNA obtained from eIF5B domains II and III, respectively, mapped onto corresponding regions of 16S rRNA. Note that eIF5B domain 4 is in front of, but does not contact the small ribosomal subunit. (**B**) The modeled position of eIF5B (in surface representation, colored purple) on the *E. coli* 70S ribosome crystal structure (adapted from [75]).

**Fig. (10):**
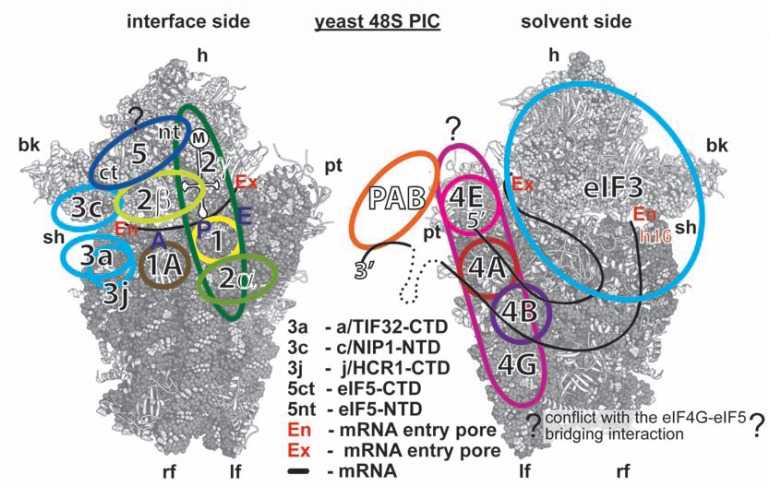
Hypothetical summary model of the structural arrangement of the yeast 48 PIC. Interface and solvent-exposed views of the tertiary structure of the 40S showing the 18S rRNA as spheres and the proteins as gray cartoons (adapted from [52]). Positions of the individual eIFs are schematically depicted as color-coded ovals based on studies referenced throughout this review. Positions with the question marks were not determined experimentally, not even proposed by structural modeling, and thus represent only the author’s best estimate.

Interestingly, although yeast eIF5B performs a number of important functions and its mutations cause severe slow growth, the gene is not essential [212], unlike IF2 [214]. The GTPase-activating center (GAC) of the large subunit is thought to induce GTP hydrolysis, however, hydrolysis of eIF5B-bound GTP is not required for the subunit joining step *per se*, but is essential for eIF5B’s own release from assembled 80S ribosomes [40,215]. In fact, the eIF5B departure from the 80S ribosomes serves as a critical check-point turning the idle – “just married” – 80S couples into actively elongating ribosomes. The structure of archaeal eIF5B revealed that it consists of four domains and has a chalice-like shape [87]. The G-domain (domain I) and domain II are homologous in both sequence and structure to the first two domains of the G proteins of the EF1A (EF-Tu) family, but domain III, although similarly positioned as domain III of EF1A, is unrelated. The C-terminal domain IV is connected to the rest of the protein by a long helix and is homologous to domain II. It was reported that a significant conformational change within eIF5B occurs upon GTP acquisition, which is required for a high-affinity interaction with the 40S subunit [39,40,215]. Actually, the position of eIF5B on the 80S ribosome has been determined using site-directed hydroxyl radical cleavage (Fig. **9**) [211], showing that it occupies the same region in the intersubunit cleft as that observed for IF2 on the bacterial 70S ribosome [216,217]. It was proposed that eIF5B, in this particular position, could interact with Met-tRNA_i_^Met ^post AUG recognition, which would help to orient eIF5B for subunit joining by burying large solvent-accessible surfaces on both subunits. Importantly, the C-terminal domain of eIF5B is known to interact with the CTT of eIF1A [76,218], which probably becomes possible only after the displacement of the eIF1A-CTT from the P-site upon AUG recognition (see above). This interaction then plays an active role in efficient subunit joining and GTP hydrolysis by eIF5B and is instrumental in the coupled dissociation of eIF1A–eIF5B from the assembled 80S ribosomes to allow the elongation phase to proceed [210,219,220].

## CONCLUSIONS AND FUTURE PERSPECTIVES

Although there has been a tremendous progress in our understanding of the molecular basis of eukaryotic translation over the last decade, it feels rather challenging when facing how many open questions still remain to be addressed. It is as if the more we learn on one front, the more unresolved issues arise on another. One of the major impediments of our further progress is undoubtedly the lack of structural information on many eIFs, with eIF3 standing first in the line, not to speak of our poor knowledge of how individual eIFs assemble, interact and “behave” on 43S and 48S PICs at different stages of translation initiation, which seem to involve rather dynamic conformational changes of the 40S subunit. Specifically, careful determination of precise positions of yeast and mammalian eIF3, eIF4F and eIF5 on the small ribosomal subunit, the high resolution structure of which is now available [52], is a rather pressing task. Undoubtedly, its resolution will greatly advance our knowledge on the molecular roles of the latter eIFs. Fig. (**10**) illustrates one of the possibilities of the structural arrangement of the yeast 48S PIC containing all eIFs based on data presented in this review and the author’s best estimate. Also, with respect to eIF3 and its roles in mRNA attachment to the 43S PIC, scanning and accuracy of AUG recognition, it will be important to investigate how eIF3 promotes these reactions on the molecular level and to determine what connections that eIF3 makes with eIF1, the eIF5-CTD, and the eIF2β-NTD in the MFC remain preserved in the scanning 48S PIC, and how they regulate the whole process. The possible roles of small ribosomal proteins and 18S rRNA elements throughout the entire mRNA binding channel in scanning and AUG selection similarly await detailed exploration.

Recently, extremely valuable tools have been developed such as, for example, ribosomal profiling [221] and single molecule assays with fluorescently tagged factors [222]. On one hand, they should be very useful to make comprehensive and quantitative ”large-scale“ measurements of translation efficiency in any organism under any conditions. On the other hand, they will enable us to detect and kinetically characterize “small-scale” rearrangements that occur in the PIC during scanning and upon AUG recognition. Besides these new approaches, it is certain that all well-established methods of yeast genetics and biochemistry as well as thermodynamic and kinetic analysis in yeast and mammalian *in vitro *systems, which recapitulate all steps of translation with purified factors, will continue to uncover many unknown aspects of the pathway. Without a doubt, it is necessary to expand our knowledge gained from yeast genetics, and yeast and mammalian *in vitro *systems into living higher eukaryotic cells. This unavoidable task brings a lot of uncertainties and potential risks of failure to set up all necessary tools to study mammalian translation *in vivo *in a comprehensive manner. The still improving methods of RNA interference and antisense approaches to knock down specific proteins should help a great deal in tackling this vastly unexplored field of supreme interest to human health. Finally, in the light of the recent surprising findings that mammalian eIFs 1, 1A and 3 play critical roles in ribosomal recycling, at least in a test tube [2], it will be of great significance to corroborate these findings also *in vivo*.
